# Carbon Negative Synthesis of Amino Acids Using a Cell-Free-Based Biocatalyst

**DOI:** 10.1021/acssynbio.4c00359

**Published:** 2024-11-21

**Authors:** Shaafique Chowdhury, Ray Westenberg, Kimberly Wennerholm, Ryan A.L. Cardiff, Alexander S. Beliaev, Vincent Noireaux, James M. Carothers, Pamela Peralta-Yahya

**Affiliations:** † School of Chemical & Biomolecular Engineering, Georgia Institute of Technology, Atlanta, Georgia 30332, United States; ‡ Bioengineering Program, Georgia Institute of Technology, Atlanta, Georgia 30332, United States; § School of Chemistry and Biochemistry, Georgia Institute of Technology, Atlanta, Georgia 30332, United States; ∥ Molecular Engineering & Sciences Institute and Center for Synthetic Biology, University of Washington, Seattle, Washington 98195, United States; # School of Physics and Astronomy, University of Minnesota, Minneapolis, Minnesota 55455, United States; ¶ Department of Chemical Engineering, University of Washington, Seattle, Washington 98195, United States; ⬢ Environmental Molecular Sciences Division, Pacific Northwest National Laboratory, Richland, Washington 99354, United States; △ Centre for Agriculture and the Bioeconomy, School of Biological and Environmental Sciences, Queensland University of Technology, Gardens Point Campus, P.O. Box 2434, Brisbane 4001, Queensland, Australia

**Keywords:** carbon negative synthesis, value-added chemicals, cell-free expression systems, metabolic engineering

## Abstract

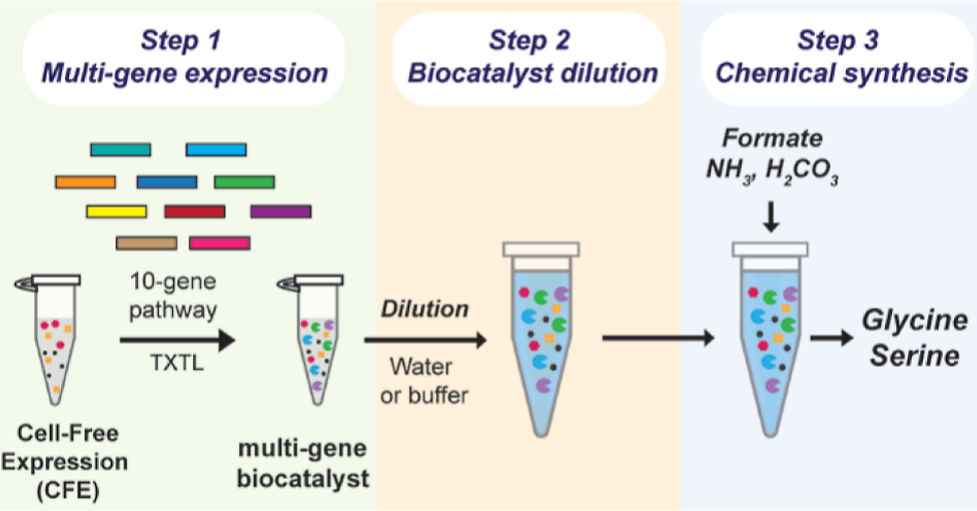

Biological systems can directly upgrade carbon dioxide (CO_2_) into chemicals. The CO_2_ fixation rate of autotrophic organisms, however, is too slow for industrial utility, and the breadth of engineered metabolic pathways for the synthesis of value-added chemicals is too limited. Biotechnology workhorse organisms with extensively engineered metabolic pathways have recently been engineered for CO_2_ fixation. Yet, their low carbon fixation rate, compounded by the fact that living organisms split their carbon between cell growth and chemical synthesis, has led to only cell growth with no chemical synthesis achieved to date. Here, we engineer a lysate-based cell-free expression (CFE)-based multienzyme biocatalyst for the carbon negative synthesis of the industrially relevant amino acids glycine and serine from CO_2_ equivalents—formate and bicarbonate—and ammonia. The formate-to-serine biocatalyst leverages tetrahydrofolate (THF)-dependent formate fixation, reductive glycine synthesis, serine synthesis, and phosphite dehydrogenase-dependent NAD(P)H regeneration to convert 30% of formate into serine and glycine, surpassing the previous 22% conversion using a purified enzyme system. We find that (1) the CFE-based biocatalyst is active even after 200-fold dilution, enabling higher substrate loading and product synthesis without incurring additional cell lysate cost, (2) NAD(P)H regeneration is pivotal to driving forward reactions close to thermodynamic equilibrium, (3) balancing the ratio of the formate-to-serine pathway genes added to the CFE is key to improving amino acid synthesis, and (4) efficient THF recycling enables lowering the loading of this cofactor, reducing the cost of the CFE-based biocatalyst. To our knowledge, this is the first synthesis of amino acids that can capture CO_2_ equivalents for the carbon negative synthesis of amino acids using a CFE-based biocatalyst. Looking ahead, the CFE-based biocatalyst process could be extended beyond serine to pyruvate, a key intermediate, to access a variety of chemicals from aromatics and terpenes to alcohols and polymers.

## Introduction

In August 2024, the atmosphere had ∼423 ppm of carbon dioxide (CO_2_), a 9% increase since 2010.^1^ Increases in CO_2_, a greenhouse gas, are associated with rising global temperatures and ocean acidification, negatively impacting human lives and biological systems.^2^ Multiple avenues are being explored toward net-zero CO_2_ emissions, including mitigating the release of CO_2_, directly capturing CO_2_ from the environment and storing it in underground geological structures, or using it as a feedstock for chemical production.^3^

Microbes have long been engineered to convert sugars, and more recently, lignocellulosic biomass, into fuels and chemicals.^4^ The food versus fuel dilemma limits the expansion of using sugars as a feedstock, while the high cost of lignocellulosic biomass deconstruction^5^ limits the economic viability of synthesizing low-cost chemicals from this renewable resource. Biologically upgrading “free” CO_2_ into products could enable the economically viable synthesis of fuels and large-volume chemicals. The CO_2_ could be from point sources, such as flue gas from steel mills (20–30 mol %) and refineries (30–40 mol %)^6^ or could be atmospheric (0.04 vol %). Electrons from solar panels or wind farms could be used to electrochemically reduce CO_2_ to formate,^7^ which now reaches more than 70% Faradaic efficiency,^7^ thus making formate a potentially viable substrate at the industrial scale. With a solubility of 97.2 g/100 mL, formate is a more biologically accessible form of carbon than CO_2_^8^ (0.17 g/100 mL) or bicarbonate (8.2 g/100 mL).

Autotrophic organisms have been engineered to convert CO_2_ into value added chemicals,^8^ including at the commercial scale. For example, LanzaTech uses engineered *Clostridia* spp. to produce ethanol from steel mill gas.^9^ Challenges with engineering organisms that naturally fix CO_2_ include (1) slow growth rate (cyanobacteria’s growth rate is 5 times slower than *Escherichia coli*), (2) low CO_2_ fixation rate (cyanobacteria achieves 5 mg/L/h while 10 mg/L/h is needed for industrial applications^8^), and (3) limited engineering of tailoring metabolic pathways to convert central carbon intermediates into value-added chemicals^10^ when compared to the biotechnology workhorse chassis *E. coli*.^11^

*E. coli*’s fast growth rate, extensive synthetic biology tools, and experimental knowledge on the optimization of hundreds of metabolic pathways^11^ have made it an attractive chassis to refactor natural^12^ and engineered synthetic^13^ CO_2_ fixation pathways.^14,15^ To date, 4 natural and 12 synthetic formate fixation pathways have been identified,^16^ with two of the synthetic pathways having been implemented in microbes.^13,15,17^ Among them, the low energy (2 ATPs), cofactor (4 NAD(P)Hs), and enzyme (9) requirements of the tetrahydrofolate (THF)-dependent formate fixation/reductive glycine synthesis (rGS) (THF/rGS) pathway make it the most energetically favorable and succinct pathway to engineer for formate upgrading.^8^ Indeed, the THF/rGS pathway has been engineered in *E. coli*,^13,15,17^*Saccharomyces cerevisiae*,^18^ and *Komagataella phaffi*(19) to drive cell growth. Due to the low formate fixation rates, doubling times are slow (66 h rather than 30 min in the case of *E. coli*(20)), with limited chemical synthesis observed.^20^

While living organisms must route some of the fixed carbon to cell growth and maintenance, nonliving biocatalysts could route 100% of the fixed carbon to chemicals synthesis. Using purified enzyme systems, the artificial starch anabolic pathway,^21^ the THF/rGS pathway,^22−24^ the crotonyl-CoA/ethylmalonyl-CoA/hydroxybutyryl-CoA (CETCH) cycle,^25^ the tartronyl-CoA pathway^26^ and the reductive glyoxylate/pyruvate cycle/malyl-CoA-glycerate (rGPS/MCG) pathway^27^ have been constructed. Specifically, the THF/rGS pathway achieved 22% conversion of formate into glycine^23^ in the presence of excess formate. Although purified enzyme systems offer exquisite control over the enzyme ratios, the cost involved in multienzyme purification will likely limit the scale up of this strategy for large-volume low-cost chemicals.

Unpurified multienzyme biocatalysts could route up to 100% of the fixed carbon to chemical synthesis while keeping the process cost down to enable the economically viable synthesis of industrial chemicals. Such biocatalysts can be generated on demand by direct expression of biosynthetic pathway genes in a nonliving lysate-based cell-free expression (CFE) and used without purification for chemical synthesis. Importantly, CFE-based biocatalysts could work over a wider range of pHs, solvents, and temperatures than microbial catalysts. Additionally, unlike microbial biocatalysts, CFE-based biocatalysts could enable product formation at maximal velocity as there are no membranes to limit substrate or product diffusivity.^28^ Briefly, lysate-based CFEs are composed of microbial cell lysate supplemented with energy compounds and reducing equivalents to support *in situ* DNA transcription and translation.^29^ Previously, individual pathway genes have been overexpressed in *E. coli* to generate enriched cell lysates and mixed-and-matched to rapidly prototype biosynthetic pathways to convert glucose into 2,3-butanediol,^30^*n*-butanol,^31^ polyhydroxyalkanoates,^32^ and mevalonate^33^ with extrapolated biosynthetic productivities (g/L/h) that often surpassed those achieved in living cells.^33^ Direct expression of pathway genes in CFE for multienzyme biocatalyst generation and use without purification has been applied to the synthesis of *n*-butanol^31^ from glucose by coexpressing 5 genes. A more common strategy, however, has been the individual expression of pathway genes in a different CFE reaction to generate individual biocatalysts, followed by mixing them together to establish the pathway. This is the case with the synthesis of 3-hydroxybuterate^34^ (2 genes), *n*-butanol^34^ (5 genes), hexanoic acid^35^ (5 genes), limonene^36^ (9 genes), and azido-sialoglycoproteins^37^ (4 genes). In general, CFE-based biocatalysts have relied on the endogenous CFE metabolism to convert glucose into central metabolic intermediates (e.g., acetyl-CoA) and regenerate cofactors (NAD(P)H) and energy equivalents (ATP). The only exception is the two-step CFE-based synthesis of styrene from phenylalanine.^38^

**Figure 1:**
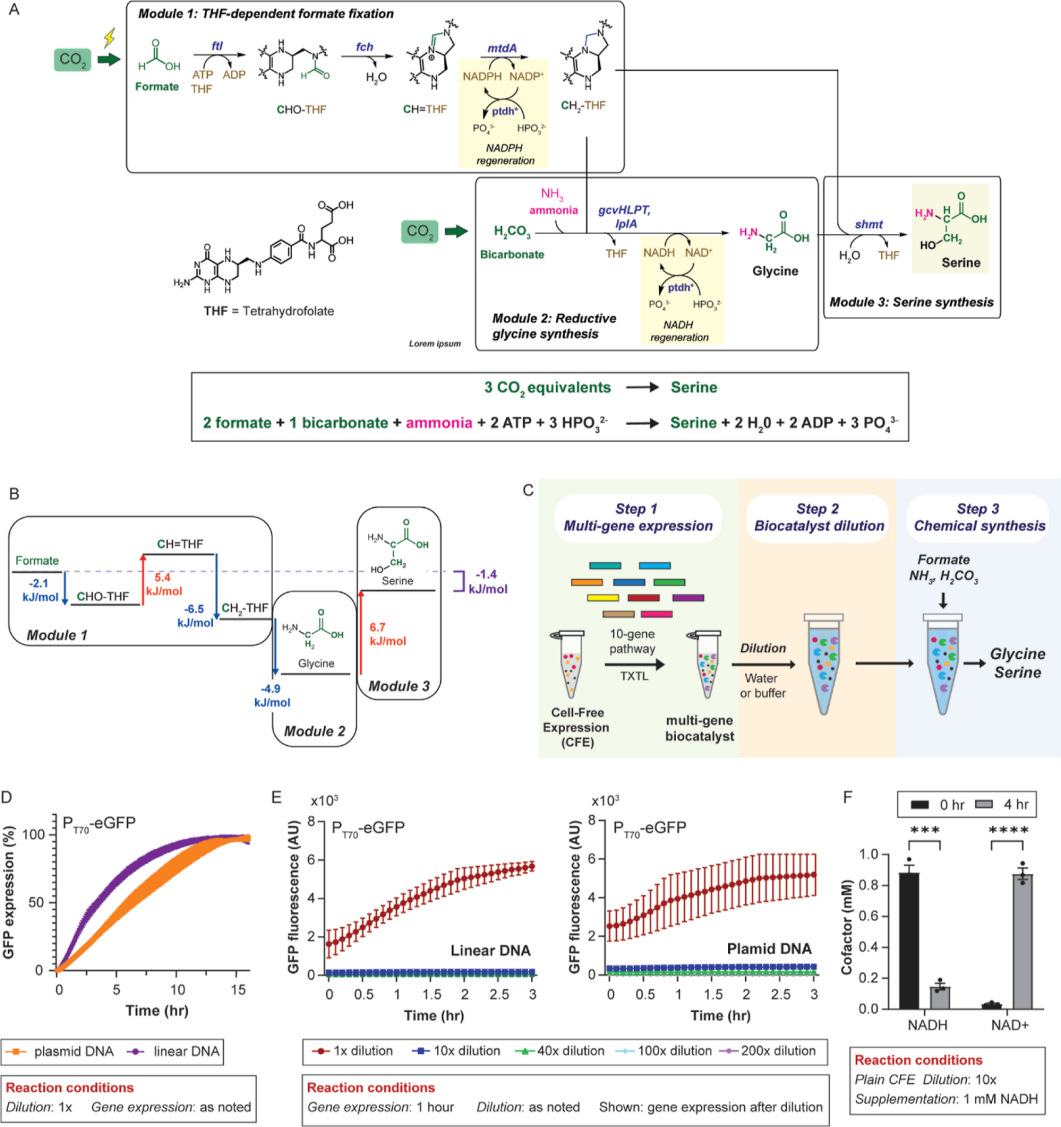
CFE-based biocatalyst for the synthesis of serine from CO_2_equivalents and ammonia. Schematic of the CFE-based 10-enzyme biocatalyst for the synthesis of serine from CO_2_ equivalents—formate and bicarbonate—and ammonia. Enzyme abbreviations in blue: *ftl*, formate-tetrahydrofolate ligase; *fch*, methenyltetrahydrofolate cyclohydrolase; *mtdA*, methylenetetrahydrofolate dehydrogenase (NADP^+^); *gcvHLPT* glycine cleavage system H, L, P and T proteins; *lplA*, lipoate-protein ligase, *shmt*, serine hydroxymethyltransferase, ptdh*, phosphite dehydrogenase mutant. Metabolite abbreviations: THF, tetrahydrofolate; CHO-THF, 10-formyltetrahydrofolate; CH=THF, 5,10-methenyltetrahydrofolate; and CH_2_–THF, 5,10-methylenetetrahydrofolate. (B) Thermodynamics for formate-to-serine synthesis. The Δ*G*′° of each step was calculated using eQuilibriator^40^ assuming a standard concentration of 1 mM for all reactants. (C) The CFE-based biocatalyst process consists of three steps: one-pot multigene expression, biocatalyst dilution, and chemical synthesis. (D) CFE of P_T70_-GFP from linear (5 nM) or plasmid (5 nM) DNA over time. (E) CFE of P_T70_-GFP from linear (5 nM) or plasmid (5 nM) is expressed for 1 h under nondiluted conditions. The reaction is then either not diluted (1×) or diluted 10×–200×. Expression of green fluorescent protein (GFP) is tracked for the next 3 h. (F) 10-fold diluted plain CFE, i.e., CFE expressing no genes, is spiked with 1 mM NADH (*t* = 0 h). The concentrations of NADH and NAD^+^ are measured after four h (*t* = 4 h). Experiments in (D–F), the lines or bars represents mean ± standard error of mean (SEM), *n* = 3.

Here, we engineer a CFE-based multienzyme biocatalyst for use without purification for the *de novo* synthesis of serine and glycine from CO_2_ equivalents—formate and bicarbonate—and ammonia (Figure 1). Serine, an industrial chemical and animal feed, has an annual global production of 350 MT/year with fermentation being the preferred production process.^39^ Glycine is a building block for the synthesis of a variety of chemicals, including herbicides and insecticides, and has an annual global production of 22,000 MT/year.^39^ Specifically, a lysate-based *E. coli* CFE is used to express a 10-gene formate-to-serine pathway in a single pot reaction to generate the formate-to-serine biocatalyst*in situ*. The multienzyme biocatalyst is composed of tetrahydrofolate (THF)-dependent formate fixation (Module 1), rGS (Module 2), and serine synthesis (Module 3). The cofactor THF is regenerated by Modules 2 and 3, while NADH and NADPH are recycled using a previously engineered bifunctional phosphite dehydrogenase mutant. One ATP is used per formate fixed, and ATP is not recycled by the system. The 10-enzyme formate-to-serine biocatalyst is diluted with an inexpensive buffer to enable higher substrate loading. After dilution, the biocatalyst is supplemented with substrates and cofactors to initiate the glycine/serine synthesis. We find that (1) the CFE-based biocatalyst is active even after 200-fold dilution, enabling higher substrate loading and product synthesis without incurring additional cell lysate cost, (2) NAD(P)H regeneration is pivotal to driving forward reactions close to thermodynamic equilibrium, (3) balancing the ratio of the formate-to-serine pathway genes loaded onto the CFE is key to improving amino acid production, and (4) efficient THF recycling enables lowering the loading of this cofactor, reducing the cost of the CFE-based biocatalyst. To our knowledge, this is the first carbon negative synthesis of amino acids using CO_2_ equivalents via a CFE-based biocatalyst. The CFE-based biocatalyst surpasses the 22% carbon conversion achieved by the rGS pathway using a purified enzyme system^23^ and the engineered rGS pathway in *E. coli* where the output was cell growth.^13,15,17^

## Results

### CFE-Based Biocatalyst for the Synthesis of Serine from CO_2_ Equivalents and Ammonia

The 10-gene formate-to-serine pathway was divided into three modules. Module 1, THF-dependent formate fixation, attaches the C1 from formate to THF to generate the C1 donor CH_2_–THF using 1 ATP and 1 NADPH in the process. Module 2, rGS, combines CH_2_–THF with bicarbonate (H_2_CO_3_) and ammonia (NH_3_) to form glycine using 1 NADH and recycling 1 THF in the process. Module 3, serine synthesis, uses a second molecule of CH_2_–THF to convert glycine to serine, recycling a second THF in the process. As both formate and bicarbonate can be directly obtained from CO_2_, synthesis of glycine captures two CO_2_ equivalents, while serine synthesis captures three CO_2_ equivalents per molecule formed (Figure 1A).

### Thermodynamics of the Formate-to-Serine Biocatalyst

The formate-to-serine biocatalyst is marginally thermodynamically favorable at Δ*G*°′ = −1.4 kJ/mol^40^ (Figure 1B). Given the lack of thermodynamic sink, cofactor regeneration (NADPH/NADH/THF) is important to drive the carbon flux forward through the pathway. While Modules 2 and 3 recycle THF, we conceive NAD(P)H regeneration as an independent unit of operation. For NAD(P)H regeneration, we first evaluated formate dehydrogenase (*fdh*) that uses formate as the electron source. As *fdh* releases one CO_2_ per NAD(P)H regenerated, we also evaluated NAD(P)H regeneration by a previously engineered phosphite dehydrogenase (ptdh*) that uses phosphite as the electron source. The ptdh*-dependent NAD(P)H regeneration would enable the use of formate as the only carbon source.

### Volumetric Expansion Strategy to Increase Product Levels Synthesized by the CFE-Based Biocatalyst

A major challenge to scaling up a CFE-based multienzyme biocatalyst for the synthesis of large-volume low-cost chemicals is the high cost of the cell lysate (∼$90/L^41^) when compared to microbial-based catalysts.^28^ Toward addressing this challenge, we dilute the biocatalyst after the gene expression step to enable greater substrate loading and increase chemical synthesis without incurring additional cell lysate cost. Figure 1C shows the steps involved in the CFE-based biocatalyst process. Step 1, gene expression, consists of expressing the multigene pathway in a one-pot CFE reaction to generate the multienzyme biocatalyst. During the gene expression step, the transcription–translation conditions optimal in the CFE system^42^ are maintained; specifically, the ratio of the cell lysate, energy molecules, and cofactors to buffer. In Step 2, biocatalyst dilution, the unpurified CFE-based biocatalyst is diluted with water or an inexpensive buffer to volumetrically expand the reaction. Step 3, chemical synthesis, sees the CFE-based biocatalyst supplemented with substrates and cofactors to initiate the chemical synthesis of the desired product. Step 2, volumetric expansion of the CFE-based biocatalyst, (1) dilutes endogenous CFE reactions, reducing the siphoning of pathway intermediates to other fates, (2) enables greater substrate loading, and (3) if the biocatalyst maintains high conversion efficiency, achieves higher chemical synthesis levels. Volumetric expansion of the formate-to-serine biocatalyst is possible because the biocatalyst does not rely on endogenous CFE reactions and regenerates its own cofactors, except for ATP. If volumetric expansion does enable greater chemical synthesis levels, it could significantly reduce bioproduction costs as no additional cell lysate would be needed to achieve higher product levels, which is key for the eventual scale up of the CFE-based process.

### Understanding the CFE-Based Biocatalyst Process

First, we set out to determine the type of DNA that could be used to generate a CFE-based biocatalyst. We tested GFP expression from a P_T70_ promoter in either plasmid (5 nM) or linear (5 nM) DNA. Both DNA forms reach GFP expression saturation at ∼16 h (Figure 1D). Thus, both linear and plasmid DNA can be used to make a CFE-based biocatalyst. Next, we hypothesized that CFE-based biocatalyst dilution alone could stop gene expression, which would bypass the need to add gene expression inhibitors that could inhibit the biocatalyst function. Specifically, after CFE-based biocatalyst dilution, the optimal TX/TL concentration (75% of the reaction) would no longer be present, thus limiting gene expression. To test this hypothesis, after 1 h of GFP expression, we either did not dilute the CFE-based biocatalyst (1× dilution) or diluted it from 10× to 200× using buffer. As shown in Figure 1E, in the nondiluted CFE-based biocatalyst, GFP continues to express for the next 3 h. However, in the 10×–200× diluted CFE-based biocatalyst, GFP expression is halted. Thus, biocatalyst dilution is sufficient to halt CFE-based gene expression. Finally, we measure the extent to which dilution would reduce the level of CFE background reactions, such as NADH consumption. To do this, we spiked 1 mM NADH to a 10× diluted plain CFE, i.e. CFE expressing no pathway genes, and measured both NADH and NAD^+^ concentrations after 4 h. As shown in Figure 1F, CFE background reactions reduced NADH to 0.15 ± 0.02 mM with the concentration of NAD + increasing to 0.88 ± 0.04 mM. Therefore, NADH oxidation by CFE background reactions is still significant even after a 10× dilution.

### Module 1: Tetrahydrofolate-Dependent Formate Fixation

**Figure 2:**
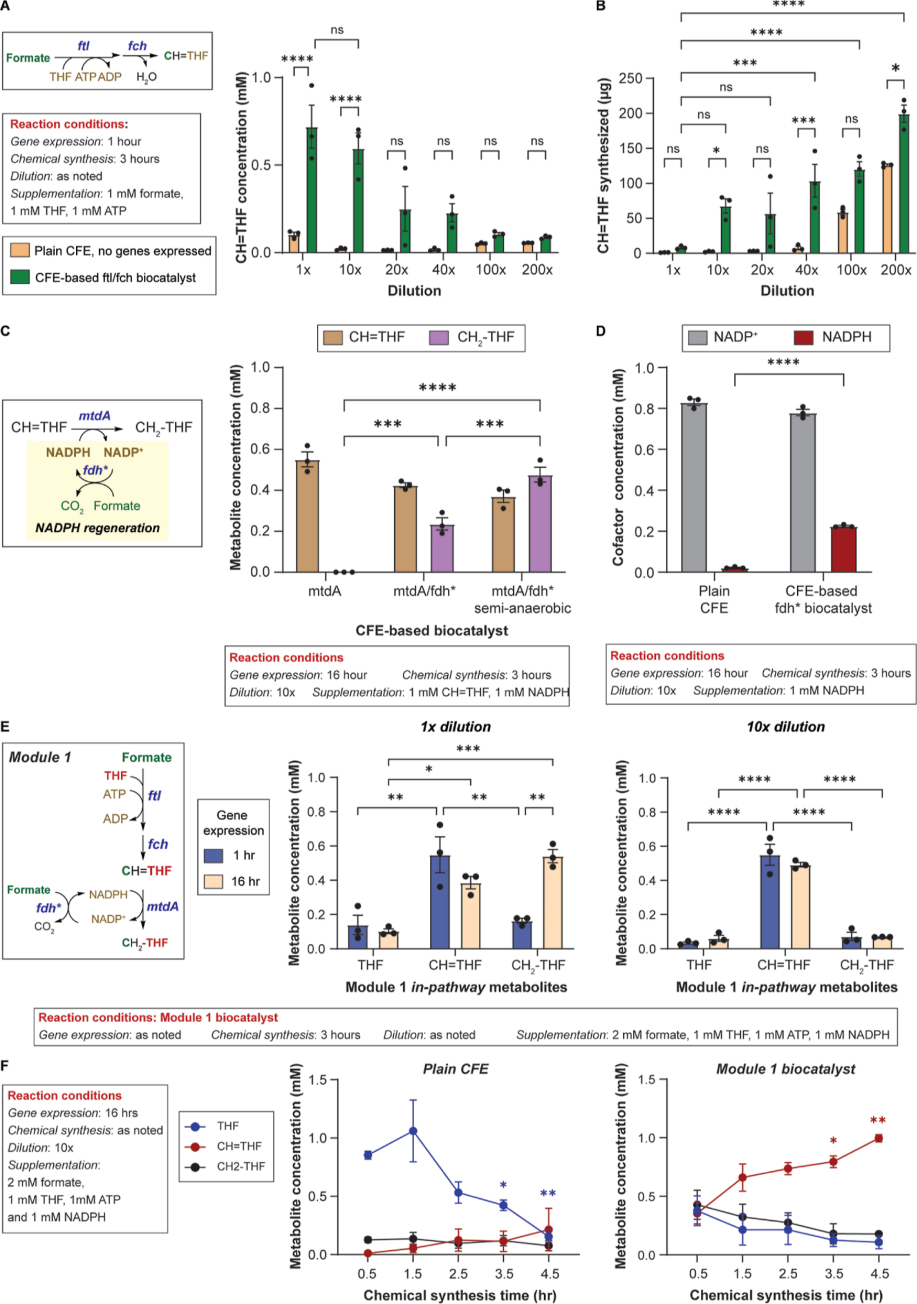
Module 1: Tetrahydrofolate-dependent formate fixation. (A,B) CFE-based *ftl*/*fch* biocatalyst performance. Top: Schematic of the reactions analyzed. (A) Conversion of formate to CH=THF by the CFE-based *ftl*/*fch* biocatalyst as a function of biocatalyst dilution. (B) Total CH=THF was synthesized by the CFE-based *ftl*/*fch* biocatalyst as a function of biocatalyst dilution. The bars represent mean ± SEM, *n* = 3, ****P < 0.0001, ***P < 0.001, *P < 0.05, ns = not statistically significant. Data were analyzed using two-way ANOVA followed by multiple comparisons via the Tukey method. (C) CFE-based *mtdA*/*fdh* biocatalyst performance. Left: Schematic of the reactions analyzed. Right: Conversion of CH=THF to CH_2_–THF by CFE-based *mtdA*, *mtdA*/*fdh*, and *mtdA*/*fdh* biocatalyst. (D) Conversion of NADP^+^ to NADPH by plain CFE, i.e., CFE not expressing pathway genes, and CFE-based fdh* biocatalyst. The bars represent mean ± SEM, *n* = 3, ****P < 0.0001, ***P < 0.0005. Data were analyzed using a two-way ANOVA followed by multiple comparisons via the Tukey method. (E) Module 1 biocatalyst performance. Left: Schematic of the reactions analyzed. Center/Right: Quantification of Module 1 in-pathway metabolites by the Module 1 biocatalyst at 1× (center) or 10× (right) dilution as a function of gene expression time. The bars represent mean ± SEM, *n* = 3, ****P < 0.0001, ***P = 0.001, **P < 0.005, *P < 0.05. Data were analyzed using a two-way ANOVA followed by multiple comparisons via the Tukey method. (F) Module 1 biocatalyst performance over time. Left: Reaction conditions. Center/Right: Quantification of Module 1 in-pathway metabolites in plain CFE (center) or the Module 1 biocatalyst (right) as a function of chemical synthesis time. Data points represent mean ± SEM, *n* = 2. The *P* values (*P < 0.05, **P < 0.005) are a comparison of either THF concentration at 0.5 h versus 3.5 or 4.5 h (blue) or CH=THF concentration at 0.5 h versus 3.5 or 4.5 h (red). Data were analyzed using a two-way ANOVA followed by a multiple comparisons via the Tukey method.

Module 1 leverages *Methylobacterium extorquens* formate-THF ligase (*ftl*), methenyl-THF cyclohydrolase (*fch*), and methylene THF dehydrogenase (*mtdA*) to fix formate to THF and ultimately generate CH_2_–THF. Formate is known to spontaneously condense with THF at pH ∼ 7 to generate CHO-THF, which can nonenzymatically cyclize to CH=THF. Therefore, we expected to see some CH=THF formed by plain CFE, i.e., CFE not expressing pathway genes. As Figure 2A shows, undiluted (1×) plain CFE supplemented with 1 mM formate, 1 mM THF, and 1 mM ATP resulted in 0.10 ± 0.01 mM CH=THF. A key advantage of a CFE-based biocatalyst is that it can be made on demand. With the goal of minimizing the CFE-based biocatalyst generation, we evaluated the CFE-based *ftl*/*fch* biocatalyst, i.e., CFE expressing *ftl* and *fch*, using a 1 h gene expression step. To determine how much the CFE-biocatalyst could be diluted while retaining activity, we diluted the *ftl*/*fch* biocatalyst from 10 to 200× using water. After dilution, we supplemented the biocatalyst with 1 mM formate, 1 mM THF, and 1 mM ATP, and ran the chemical synthesis step for 3 h. If successful, CH=THF could be generated from formate in as little as 4 h. As shown in Figure 2A, the undiluted *ftl*/*fch* biocatalyst resulted in 0.72 mM ± 0.12 mM CH=THF, while the 10× diluted *ftl*/*fch* biocatalyst resulted in statistically the same concentration at 0.60 mM ± 0.09 mM CH=THF. Taken together, the rate of formate-to-CH=THF conversion can only be maintained in up to a 10× biocatalyst dilution. Beyond that, the biocatalyst is too diluted to operate at the same conversion rate as the nondiluted biocatalyst.

However, the power of CFE-based biocatalyst dilution rests in the potential for a higher substrate loading to achieve higher product levels. In terms of total mass of CH=THF produced, 40×, 100× and 200× diluted *ftl*/*fch* biocatalyst produce statistically higher mass of CH=THF than the undiluted *ftl*/*fch* biocatalyst (Figure 2B). Interestingly, in 100× and 200× diluted plain CFE, we observe high spontaneous synthesis of CH=THF, 49% and 63% of the total CH=THF produced by 100× and 200× diluted *ftl*/*fch* biocatalyst, respectively. After removing the contribution of spontaneous CH=THF synthesis, we find that the 200× diluted *ftl*/*fch* biocatalyst results in 10-fold more CH=THF (73.41 ± 12.45 μg) than the undiluted *ftl*/*fch* biocatalyst (6.99 ± 1.41 μg). Therefore, volumetric expansion of the *ftl*/*fch* biocatalyst enabled higher CH=THF synthesis levels.

Next, we evaluated the NADPH-dependent reduction of CH=THF to CH_2_–THF (Figure 2C). Given the similar chemical conversion rates of the 1× and 10× diluted *ftl*/*fch* biocatalyst, we evaluated the performance of the CFE-based *mtdA* biocatalyst at 10× dilution only. As GFP expression reaches saturation after ∼16 h (Figure 1D), we set the gene expression step for 16 h, while keeping the chemical synthesis step at 3 h. As shown in Figure 2C, the 10× diluted *mtdA* biocatalyst supplemented with 1 mM CH=THF and 1 mM NADPH did not result in detectable levels of CH_2_–THF. As CH=THF reduction to CH_2_–THF is near thermodynamic equilibrium (Figure 1B), we introduced the *in situ* regeneration of NADPH to help replenish the NADPH pool and drive the reaction forward. Toward this goal, we used an engineered *A. thaliana* formate dehydrogenase (fdh*: *fdh*:D227Q/L229H), which has been previously used for NADPH regeneration.^43^ The 10× diluted *mtdA*/fdh* biocatalyst supplemented with 1 mM CH=THF and 1 mM NADPH resulted in 0.24 mM ± 0.03 mM CH_2_–THF. As *mtdA* is known to be oxygen-sensitive,^44^ we evaluated the 10× diluted *mtdA*/fdh* biocatalyst under semianaerobic conditions, which resulted in 0.48 mM ± 0.04 mM CH_2_–THF.

To confirm that fdh* was regenerating NADPH, we evaluated the performance of a 10× diluted CFE-based P_T70_-fdh* biocatalyst supplemented with 1 mM NADP^+^ (Figure 2D). The 10× fdh* biocatalyst results in a statistically higher NADPH regeneration when compared with the 10× diluted plain CFE control. Taken together, the 10× diluted CFE-based *mtdA* biocatalyst converts CH=THF to CH_2_–THF, and its yield is enhanced by regenerating NADPH and employing semianaerobic reaction conditions.

To generate the complete Module 1 biocatalyst, we expressed *ftl*, *fch*, *mtdA*, and fdh* in CFE (Figure 2E). Concerned about potential low carbon flux through the four-enzyme system, we evaluated the Module 1 biocatalyst at both 1× and 10× dilution while keeping the chemical synthesis step constant at 3 h. We assessed gene expression steps of 1 and 16 h to gain insight into how fast the Module 1 biocatalyst could be generated. As shown in Figure 2E, the nondiluted Module 1 biocatalyst using a 16 h gene expression treatment resulted in statistically significant higher CH_2_–THF (0.54 ± 0.04 mM) than using a 1 h gene expression treatment. We observed buildup of the CH=THF in the 1 h gene expression treatment hinting at *mtdA* limiting CH_2_–THF synthesis. In the 10× diluted Module 1 biocatalyst, we see CH=THF accumulation at 1 and 16 h gene expression treatments. Taken together, in the 10× Module 1 biocatalyst, *mtdA* limits the conversion of CH=THF to CH_2_–THF, with CH=THF accumulating in the system to 0.49 ± 0.01 mM.

To determine if a 3 h chemical synthesis step was optimal for CH_2_–THF synthesis, we varied the chemical synthesis step from 0.5 to 4.5 h while keeping the 16 h gene expression step constant. Both 10× diluted plain CFE and 10× diluted Module 1 biocatalyst were supplemented with 1 mM formate, 1 mM THF, 1 mM ATP, and 1 mM NADPH to initiate the chemical synthesis step. As shown in Figure 2F, in plain CFE, there is no statistically significant increase in the concentration of CH=THF or CH_2_–THF. Interestingly, the concentration of THF drops after 3.5 h. We hypothesize that THF is dissipated by the CFE background metabolism. Tetrahydrofolate could be used, among others, in the biosynthesis of thymidylate, purine, or methionine. In the case of the Module 1 biocatalyst, the concentration of THF dropped to 0.38 ± 0.13 mM after just 30 min with the concentration of CH=THF increasing over time peaking at 1.00 ± 0.03 mM after 4.5 h. To maximize CH=THF synthesis while minimizing the overall process time, we used a 4 h chemical production step in subsequent experiments. We hypothesized that successful implementation of Modules 2 and 3, both of which use CH_2_–THF as a substrate, would pull on CH=THF to be converted to CH_2_–THF as needed.

### Module 3: Serine Synthesis

**Figure 3:**
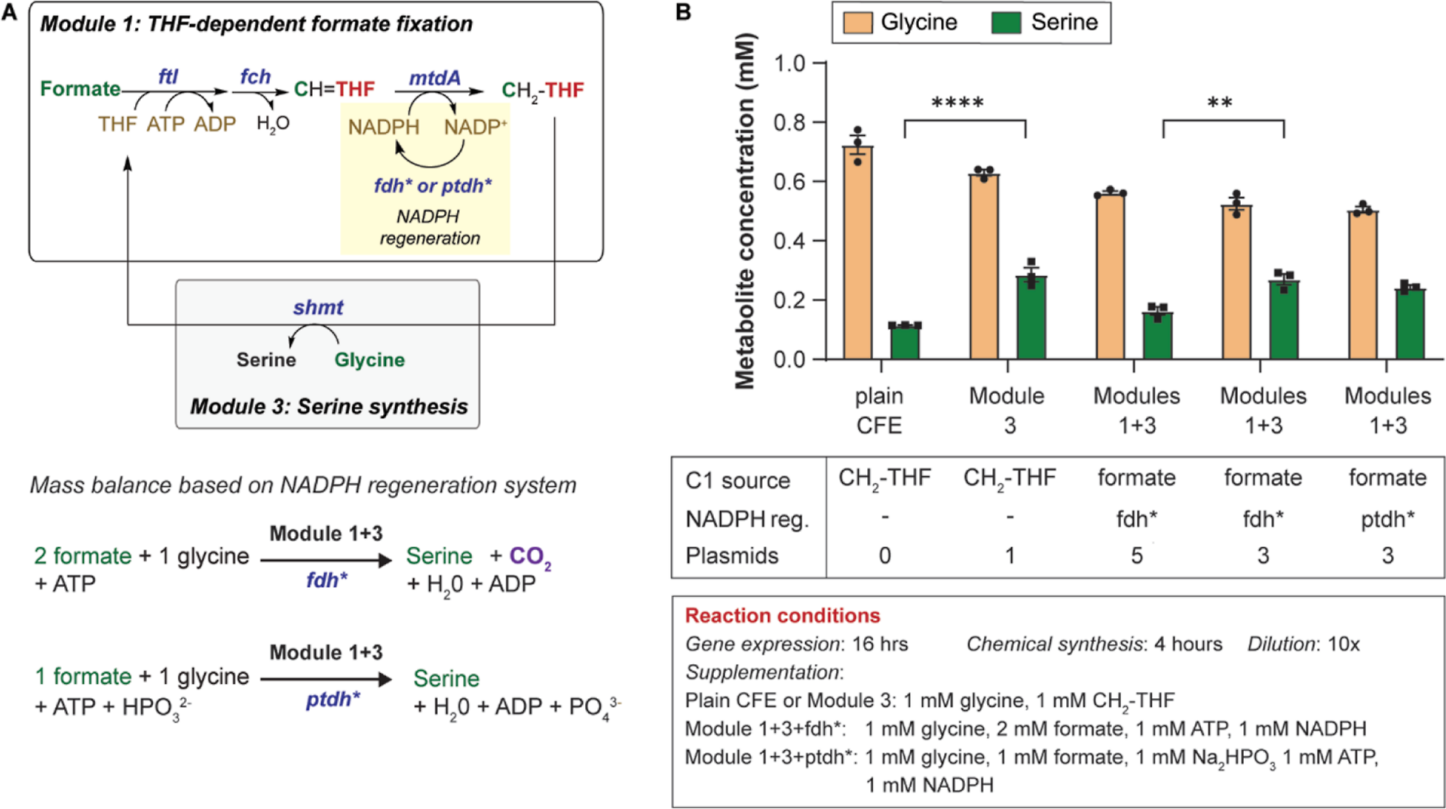
Module 3: Serine synthesis. (A) Top: Schematic of Module 1 (THF-dependent formate fixation) and Module 3 (Serine synthesis). Bottom: Mass balance of Modules 1 + 3 biocatalyst when using a formate dehydrogenase mutant (fdh*) or a phosphite dehydrogenase mutant (ptdh*) for NADPH regeneration. (B) Serine synthesized by Module 1 and Module 1 + 3 biocatalysts as a function of carbon source, NADPH regeneration system, and plasmid number. Bars represent mean ± SEM, *n* = 3, **p < 0.005, ***p < 0.0005., ****p < 0.0001. Data were analyzed using a two-way ANOVA followed by a multiple comparison via the Tukey method.

The product of Module 1, CH_2_–THF, enters both rGS (Module 2) and serine synthesis (Module 3). Due to the complexity of Module 2, requiring multiple substrates and cofactors (CH_2_–THF, NH_3_, H_2_CO_3_, NADH) to form glycine, we first investigated Module 3, which is composed of a single enzyme, *E. coli* serine hydroxymethyltransferase (*shmt*). Module 3 brings together glycine and CH_2_–THF to synthesize serine and regenerate THF in the process (Figure 3A). For all reactions, we used a 16 h gene expression step, 10× biocatalyst (or plain CFE) dilution, and a 4 h chemical synthesis step. As shown in Figure 3B, when supplemented with 1 mM glycine and 1 mM CH_2_–THF as the C1 source, 10× diluted plain CFE generates 0.11 mM ± 0.00 mM serine due to CFE background reactions, while 10× diluted Module 3 biocatalyst doubled serine synthesis, reaching 0.29 mM ± 0.02 mM. Next, we tested the ability of a 10× diluted Module 1 + 3 biocatalyst using fdh* for NADPH regeneration to use formate as the C1 source. When supplemented with 1 mM glycine and 2 mM formate (1 mM as C1 source, 1 mM for NADPH regeneration), 1 mM ATP, and 1 mM NADPH, the Module 1 + 3 biocatalyst resulted in 0.16 mM ± 0.01 mM serine.

The Module 1 + 3 + fdh* biocatalyst consists of 5 plasmids, which could create burden on the CFE. Unlike plasmid burden in cells where it is associated with plasmid replication and maintenance,^45^ plasmid burden in CFE plasmid is associated with transcriptional and translational competition,^46^ such as superfluous synthesis of antibiotic markers (β-lactamase gene in this case) that reduces the availability of transcription and translation machinery for pathway genes. To reduce CFE burden, we moved the Module 1 biocatalyst from a three-plasmid to a one-plasmid system (Figure 3B). The 3-plasmid Module 1 + 3 + fdh* biocatalyst results in significantly higher serine synthesis, reaching 0.27 mM ± 0.02 mM. Of note, it is possible that the differences in chemical synthesis may be due to differences in gene expression caused by the arrangement of the genes in an operon versus being independently expressed from a promoter.

With the goal of using formate only as a C1 source, we explored an NADPH regeneration mechanism using a *Pseudomonas stutzeri* phosphite dehydrogenase mutant (ptdh*) capable of regenerating both NADPH and NADH using phosphite as the reducing power.^47^ A Module 1 + 3 + ptdh* supplemented with 1 mM glycine and 1 mM formate, 1 mM Na_2_HPO_3_, 1 mM ATP, and 1 mM NADPH resulted in 0.24 ± 0.01 mM serine. Although not statistically different than using fdh*, we used ptdh* for NADPH regeneration going forward as it enables the use of formate only as a carbon source improving the carbon negativity of the chemical synthesis (Figure 3A). Taken together, Modules 1 + 3 + ptdh* captures one CO_2_ equivalent (formate) in the synthesis of serine from glycine. It is worth noting that both glycine and serine can be consumed by the background CFE reactions. Thus, 0.24 mM serine may be a lower limit of the serine synthesized.

### Module 2: Reductive Glycine Synthesis

**Figure 4:**
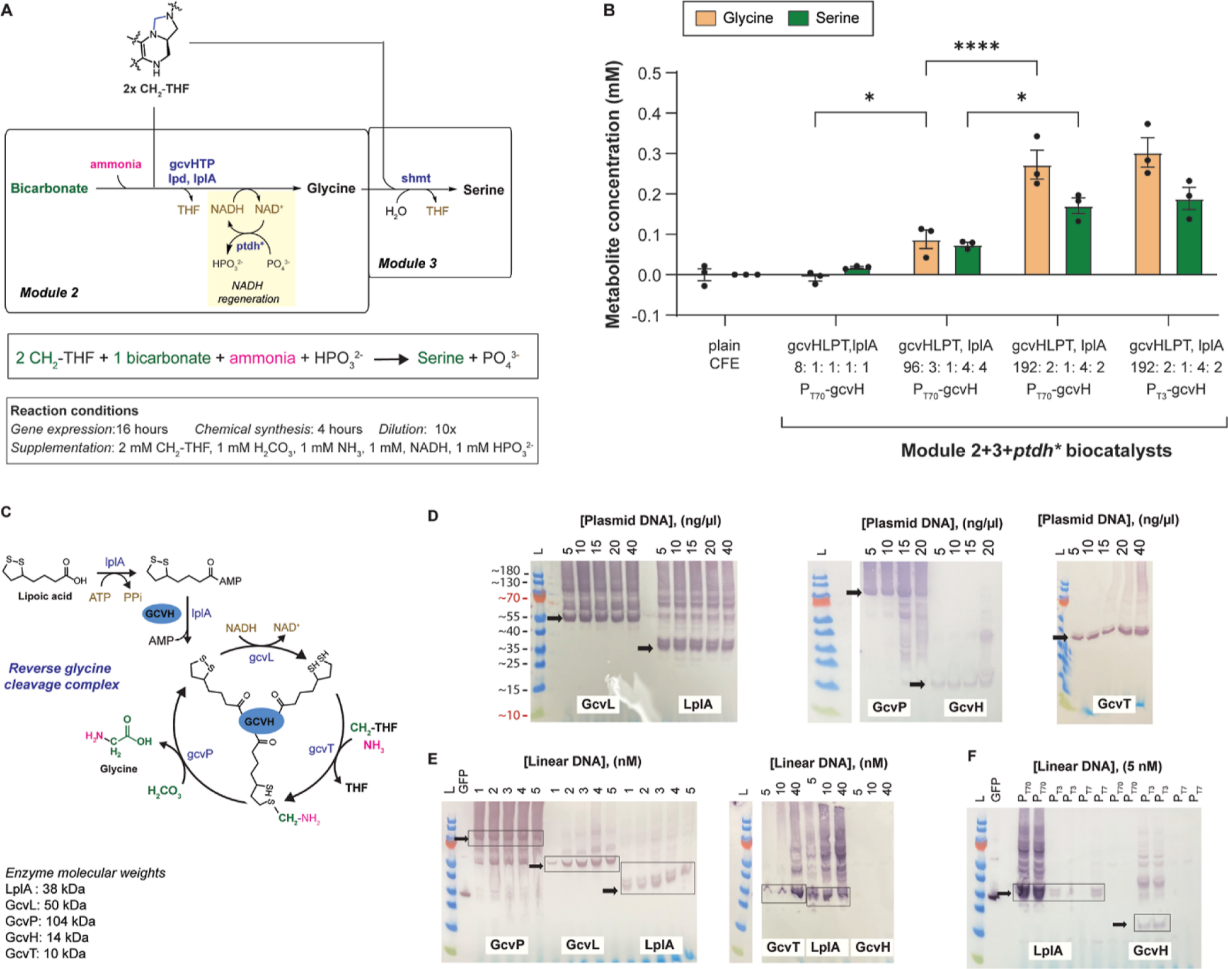
Module 2: Glycine synthesis. (A) Schematic of Module 2 and Module 3 that convert CH_2_–THF, H_2_CO_3_, NH_3_ to glycine and serine. (B) Metabolite concentration as a function of Module 2 + 3 + ptdh* biocatalyst. The bars represent mean ± SEM, *n* = 3, *P < 0.05, ****P < 0.0001. Data were analyzed using two-way ANOVA followed by a multiple comparisons via the Tukey method. (C) Enzymatic steps are involved in the reverse glycine cleavage complex. (D) Western blot showing the protein levels of Module 2 genes when using plasmid DNA. All genes were His_6_-tagged at the N-terminus driven by a P_T70_ promoter. (E) Western blot showing the protein levels of Module 2 genes when using liner DNA. All genes were His_6_-tagged at the N terminus and driven by a P_T70_ promoter. (F) Western blot showing the protein levels of *lplA* and *gcvH* when using liner DNA. The genes were His_6_-tagged at the N terminus and driven by P_T70_, P_T3_, or P_T7_ promoters.

In Module 2, the glycine cleavage complex (gcv) is run in reverse, converting CH_2_–THF, H_2_CO_3_, and NH_3_ to glycine using one NADH and recycling THF in the process. This conversion is also called reverse glycine synthesis (rGS). Module 2 is composed of the *E. coli*, glycine cleavage complex H, L, P, and T proteins (*gcvHLTP*), and lipoyl ligase (*lplA*) (Figure 4A). Although rGS has been implemented in microbes,^13,20^ unique challenges arise when moving this system to CFE. First, CFE lacks the biosynthetic pathways for lipoic acid and pyridoxal phosphate, which need to be supplemented. Second, in CFE, the *gcvHLPT* genes are expressed from the P_T70_ promoter rather than from their endogenous promoter/operon. Synthetic regulation of *gcvHLPT* expression in CFE may result in a suboptimal *gcvHLPT* enzyme ratio, which may affect the glycine synthesis. Previously, the rGS was reconstituted using purified enzymes, and the *gcvHLPT* enzyme ratio of 8:1:1:1^22^ was identified as optimal for pathway functionality. Thus, a key challenge in engineering rGS in CFE is the identification of the *gcvHLPT* gene ratio to add to the CFE for direct gene expression to approximate the 8:1:1:1 *gcvHLPT* enzyme ratio.

Given that the preferred direction of rGS is in the glycine cleavage direction, we tested Module 2 together with Module 3 to pull any glycine synthesized to serine. For all experiments, we used ptdh* for NADH regeneration, a 16 h gene expression step, 10× biocatalyst (or plain CFE) dilution, and a 4 h chemical synthesis step. To initiate chemical synthesis, we supplemented the CFE-based biocatalyst (or plain CFE) with 2 mM CH_2_–THF (one equivalent going into Module 2, the second equivalent going into Module 3), 1 mM H_2_CO_3_, 2 mM NH_3_, 1 mM NADH, and 1 mM Na_2_HPO_3_. As shown in Figure 4B, the plain CFE control did not produce glycine or serine. Since *gcvH*, *gcvL*, *gcvP*, and *gcvT* genes were expressed from the same P_T70_ promoter, we assumed similar transcription–translation levels for all four genes. A Module 2 + 3 + ptdh* biocatalyst using a *gcvHLPT* gene ratio of 8:1:1:1 did not significantly increase the concentration of glycine or serine synthesized.

Hypothesizing that *gcvHLPT* genes were not expressed at the same levels, using Western blots, we determined the relative protein levels of *gcvHLPT* and lplA, which loads lipoic acid onto GcvH (Figure 4C). Toward this goal, the *gcvHLPT* and lplA genes were His_6_-tagged at the N-terminus and detected using an anti-polyhistidine antibody. As Figure 4D shows, there is robust expression of *gcvL*, *gcvP* and *lplA*, all peaking at 5 ng/μL, and *gcvT* peaking at 20 ng/μL. Expression of *gcvH*, however, was markedly lower, and increases in plasmid concentration did not increase protein levels. Taken together, *gcvH* expression limits the modulus of the Module 2 biocatalyst.

To improve *gcvH* expression, we first investigated the use of linear DNA to achieve higher *gcvH* gene loading into the CFE. Briefly, the formate-to-serine pathway consists of seven plasmids, and further increases in plasmid concentration lead to DNA viscosity issues. Thus, we moved Module 2 to a linear DNA system and used a CFE optimized to prevent nucleic acid degradation.^48^ We then used Western blots to determine the relative protein levels of *gcvHLPT* and *lplA* (His_6_-taged at the N-terminus) when they were expressed from linear DNA (Figure 4E). Using the protein bands’ pixel intensities, we calculated the approximate ratio of *gcvP*, *gcvL*, and *lplA* to be 1:3:4 when 2–4 nM of either *gcvP*, *gcvL*, or *lplA* were used. Expression of *gcvT* and *lplA* were similar to one another, while the expression of *gcvH* was markedly lower, even at 40 nM. Next, we investigated expressing *gcvH* from the stronger P_T3_ and P_T7_ promoters. As shown in Figure 4F, P_T3_-*gcvH* leads to higher protein expression than P_T70_-*gcvH* or P_T7_-*gcvH*. Interestingly, P_T3_ did not improve the expression of other Module 2 genes such as lplA.

Using the Western blots protein bands’ pixel intensity, we calculated that to achieve a *gcvHLPT*/*lplA* protein ratio of ∼1:1:1:1:1, the *gcvHLPT*/*lplA* gene ratio loaded to the CFE ought be ∼12:3:1:4:4. Thus, to achieve a *gcvHLPT* protein ratio of 8:1:1:1, the calculated DNA molar ratio of *gcvHLPT*/*lplA* should be ∼96:3:1:4:4. As shown in Figure 4B, a Module 2 + 3 + ptdh* biocatalyst with a *gcvHLPT*/*lplA* gene ratio of 96:3:1:4:4 using P_T70_-*gcvH* results in 0.09 ± 0.02 mM of glycine. We then attempted to saturate the system with *gcvH* by doubling the concentration of P_T70_-*gcvH*. A Module 2 + 3 + ptdh* biocatalyst with a *gcvHLPT*/*lplA* gene ratio of 192:2:1:4:2 results in statistically higher concentration of amino acids with glycine at 0.27 ± 0.04 mM and serine at 0.17 ± 0.02 mM. Finally, we swapped P_T70_-*gcvH* for P_T3_-*gcvH* in the Module 2 + 3 + ptdh* biocatalyst, but that swap did not increase glycine or serine synthesis. Taken together, a Module 2 + 3 + ptdh* biocatalyst with a *gcvHLPT*/*lplA* gene ratio of 192:2:1:4:2 results in a combined 0.44 mM glycine and serine synthesis, or 44% conversion of the CH_2_–THF to amino acids.

### Formate-to-Serine Biocatalyst

We expressed the 10-gene formate-to-serine pathway (Module 1 operon, Module 2 (192:2:1:4:2, P_T3_-*gcvH*), *shmt* and ptdh*) in a single pot CFE reaction to generate the formate-to-serine biocatalyst. In this biocatalyst, ptdh* would regenerate NADPH (Module 1) and NADH (Module 2).

**Figure 5:**
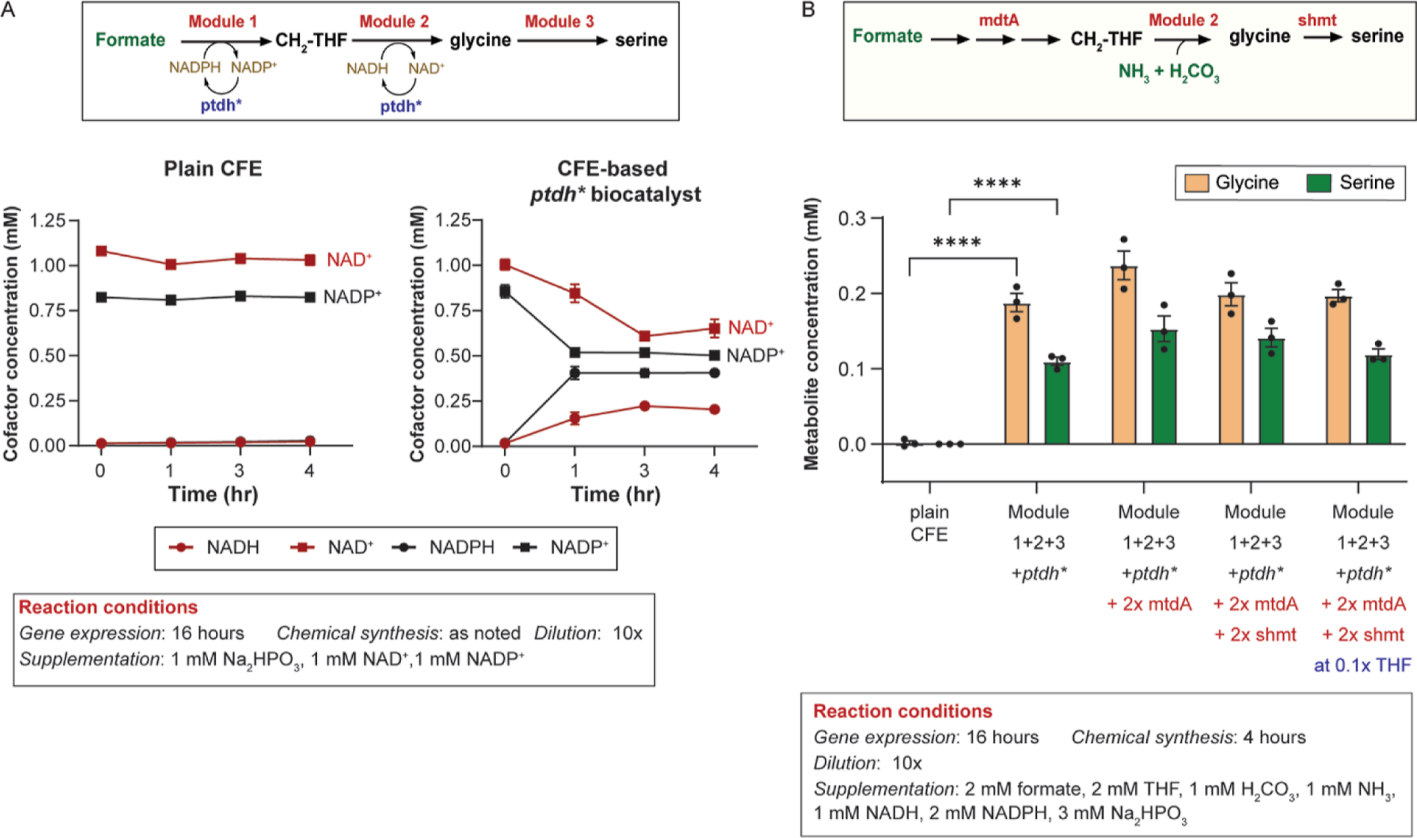
*De novo* synthesis of serine and glycine from formate, bicarbonate, and ammonia. (A) Regeneration of NADPH and NADH by P. stutzeriptdh* directly expressed in CFE. Left: Reduction of 1 mM NAD^+^ and 1 mM NADP^+^ was achieved by plain CFE. Right: Reduction of 1 mM NAD^+^ and 1 mM NADP^+^ by the CFE-based ptdh* biocatalyst. (B) Synthesis of serine and glycine from formate, bicarbonate, and ammonia. Reaction conditions are as shown. For Module 2, *gcvHLPT*/*lplA* genes were at a 192:2:1:4:2 ratio.

Thus, we first evaluated the ability of ptdh* to recycle NADPH and NADH simultaneously in the context of CFE. For the evaluation, we used a 16 h gene expression step, 10x biocatalyst (or plain CFE) dilution, and a chemical synthesis step ranging from 0 to 4 h. The ptdh* biocatalyst or plain CFE was spiked with 1 mM NAD^+^ and 1 mM NADP^+^ at the beginning of the chemical synthesis step. As shown in Figure 5A, the plain CFE control does not reduce NAD^+^ or NADP^+^ to NADH or NADPH, respectively. On the other hand, P_T70_-ptdh* directly expressed in CFE successfully reduces NAD^+^ into NADH and NADP^+^ into NADPH in as fast as 1 h. We observe that the ptdh* biocatalyst is more efficient at reducing NADP^+^ than is NAD^+^.

Next, we measured the ability of the formate-to-serine biocatalyst to synthesize serine from formate, bicarbonate, and ammonia. We used a 16 h gene expression step, a 10× biocatalyst (or plain CFE) dilution, and a 4 h chemical synthesis step. After dilution, the reaction was supplemented with 2 mM formate, 2 mM THF, 1 mM H_2_CO_3_, 1 mM NH_3_, 1 mM NADH, 2 mM NADPH, and 3 mM Na_2_HPO_3_. As shown in Figure 5B, the plain CFE control did not produce glycine or serine, while the formate-to-serine biocatalyst resulted in 0.19 ± 0.01 mM glycine and 0.11 ± 0.0 mM serine or a combined 0.3 mM glycine and serine or 30% conversion of formate to amino acids.

Knowing that *mtdA* limits conversion of CH=THF to CH_2_–THF in Module 1, and CH_2_–THF is used by both Modules 2 and 3, we attempted to increase the synthesis of glycine and serine by applying a “push” metabolic engineering strategy (Figure 5B). Specifically, we doubled the DNA concentration of *mtdA* (2× *mdtA*) in the CFE; however, it did not result in a statistically higher concentration of serine or glycine. We then attempted to reduce the buildup of the glycine intermediate by applying a “pull” strategy and doubling the DNA concentration of *shmt* (2× *shmt*) in the system. A formate-to-serine biocatalyst with 2× *mtdA* and 2× *shmt* did not statistically improve the concentration of glycine or serine. In conclusion, optimization of the formate-to-serine biocatalyst is not trivial and will require, in the future, more systematic pathway optimization strategies including design of experiments/machine learning and kinetic pathway modeling.

### CFE-Based Biocatalyst Process Cost

We were interested in determining the contribution of volumetric expansion to reducing the cost of the CFE-based formate-to-serine biocatalyst. Using literature values for the cost of CFE ($90/L^41^) and publically available data for the cost of cofactors, we determined that the cost of nondiluted (1×) CFE-based formate-to-serine biocatalyst is $1.72/mL, while a 10-fold diluted biocatalyst costs marginally less at $1.66/mL (Table 1). The small decrease in cost is because the cell lysate TX/TL only accounts for 4% of the total cost. Tetrahydrofolate is the major cost contributor, with 50% of the total cost, closely followed by NADPH at 41%. Thus, reducing the concentration of THF in the biocatalyst would have a major effect on the process cost. Toward this goal, we reduced the THF concentration 10-fold, from 2 mM to 0.2 mM, which, if successful, would halve the cost of a 10× diluted CFE-based formate-to-serine biocatalyst to $0.89/mL. As shown in Figure 5B, a formate-to-serine biocatalyst with 2× *mtdA*, 2× *shmt*, and 0.1× THF concentration results in 0.20 mM ± 0.01 mM glycine and 0.12 mM ± 0.01 mM serine, which is comparable to the glycine/serine synthesis when using 2 mM THF. Thus, Module 2 and Module 3 are efficiently recycling THF. Further reductions in CFE-based formate-to-serine biocatalyst will come from reducing the concentration of NADPH in the system.

**Table 1:** Cost of CFE-Based Formate-to-Serine Biocatalyst Components

chemical	cost ($)	conc.	1× dilution cost per 1 mL ($)	10× dilution cost per 1 mL ($)	200× dilution cost per 1 mL ($)
cell lysate-based TX/TL	90/L^41^	75% of vol	6.75 × 10^–2^	6.75 × 10^–3^	3.38 × 10^–4^
THF	970/g^a^	2 mM	0.86	0.86	0.86
ATP	33.6/g^a^	2 mM	3.70 × 10^–2^	3.70 × 10^–2^	3.70 × 10^–2^
NADH	60/g^b^	1 mM	4.26 × 10^–2^	4.26 × 10^–2^	4.26 × 10^–2^
NADPH	428/g^c^	2 mM	0.71	0.71	0.71
Na_2_HPO_3_	0.12/g^a^	3 mM	7.65 × 10^–5^	7.65 × 10^–5^	7.65 × 10^–5^
**total cost**			**1.72**	**1.66**	**1.66**
**total cost (0.1× THF)**			**0.95**	**0.89**	**0.88**

a Milipore Sigma.

b Cayman Chemicals.

c Santa Cruz Biotechnologies.

## Discussion

We successfully engineered a 10-enzyme CFE-based formate-to-serine biocatalyst that captures 3 CO_2_ equivalents (2 formates, 1 bicarbonate) and an ammonia per serine synthesized. The 30% conversion of formate to serine and glycine surpasses the previous formate to glycine conversion of 22% achieved via a reconstitution of rGS using purified enzymes.^23^ The formate-to-serine biocatalyst regenerates THF via Modules 2 and 3 and NADH and NADPH via a previously engineered bifunctional phosphite dehydrogenase mutant. The formate-to-serine biocatalyst does not regenerate ATP, which can be implemented in the future by adding a polyphosphate kinase.^49^ Of note, neither ATP nor cell lysate TX/TL is the major cost in CFE-based formate-to-serine biocatalyst, but instead, it is THF, accounting for 50% of the cost. The efficient recycling of THF by the system allows for halving the cost of the 10× diluted CFE-based formate-to-serine biocatalyst. In the future, additional reductions in the concentration of THF and NADPH cofactors in the CFE-based formate-to-serine biocatalyst could further reduce the biocatalyst cost.

When compared to traditional biocatalysts that require microbial enzyme expression followed by purification before use, CFE-based biocatalysts can be produced on-demand and *in situ* via direct expression of DNA in CFE. The ability to rapidly generate the CFE-based biocatalysts allowed the rapid screening of (1) gene expression duration, (2) biocatalyst dilutions, (3) chemical synthesis time lengths, (4) NADH regeneration systems, (5) analysis of *gcvHLPT*/*lplA* gene ratios, (6) dosing of bottleneck genes (*mtdA*, shmt), and (7) THF loading. The volumetric expansion of the CFE-based biocatalyst explored in this work (up to 200-fold) enabled a 10-fold increase in the mass of CH=THF synthesized without incurring additional cell lysate TX/TL costs. Finally, because CFE-based biocatalysts are nonliving, carbon is not diverted to cell growth and maintenance, although we do observe cofactor dissipation (NADH, THF) by CFE background reactions.

Background CFE reactions may be siphoning some of the glycine and serine synthesized as well as the NAD(P)H generated by the 10× diluted CFE-based formate-to-serine biocatalyst. Thus, the concentrations of serine and glycine measured maybe a lower limit of the amino acids synthesized. Further CFE-based biocatalyst dilution should decrease deviation of these metabolites to CFE background reactions and potentially lead to greater serine and glycine yields. Additionally, competing reactions could be knocked out in the strains used to prepare the lysate^41^ or by direct intervention with small molecule or peptide inhibitors. If thermophilic enzymes for a desired pathway can be expressed in CFE,^50^ then heat denaturation could eliminate competition from background reactions present in mesophilic *E. coli* lysate. Overall, lowering the dissipation of cofactors to the CFE background could enable the synthesis of a wide variety of industrial products^11^ with close to 100% carbon efficiency.

Finally, in this work, all pathway genes are expressed in a single-pot reaction; thus, all enzymes are generated at the same time. In the future, controlling the timing and expression levels of the 10 pathway genes could be important for achieving optimized enzyme stoichiometries for multistep biosynthetic pathways.^29^ With the CFE-based formate-to-serine biocatalyst and the analytics to measure in-pathway intermediates in hand, in the future, the rapid prototyping of CFE-based biocatalysts can be exploited to correlate pathway gene ratios to serine and glycine concentrations to generate the data needed to drive machine learning models for the further improvement of product yields. Additionally, other data-drive modeling, such as kinetic pathway modeling, could also be applied to the formate-to-serine pathway to gain additional insight.

## Methods

### Materials

All materials, including chemicals, solvents, kits, plasmids, primers, and gene sequences, can be found in the Supporting InformationTables S1–S7. Sources for key substrates, cofactors, and products: Tetrahydrofolate, 5,10-methenyl THF, 5,10-methylene THF, NADH, and NADPH were purchased from Cayman Chemicals. Formic acid was purchased from Fischer Scientific. Serine, glycine, ammonia solution in water, ATP, DTT, α-lipoic acid, catechol, sodium dihydrogen phosphate, and sodium bicarbonate were purchased from Millipore Sigma. Pyridoxal-5-phosphate was purchased from TCI Chemicals. Fmoc chloride was purchased from Oakwood Chemical. CFE system was purchased from Arbor Biosciences.

### Formate-to-Serine Pathway—Plasmid DNA

For CFE-based biocatalysis experiments, all genes were used without any tags. Genes under control of the P_T70_ promoter were cloned into pTXTL-P70a-deGFP (Arbor Biosciences)^42^ between *NdeI*/*XhoI* using Gibson assembly. Genes under control of the P_T7_ promoter were cloned into pTXTL-T7-deGFP (Arbor Biosciences) between *NcoI*/*XhoI* using a Gibson assembly. Genes under control of the P_T3_ promoter were cloned into pTXTL-T3-deGFP (Arbor Biosciences) between *NcoI*/*XhoI* using Gibson assembly. *M. extorquensftl*, *fch*, and *mtdA* as well as A. thalianafdh* (*fdh*:D227Q/L229H)^43^ were codon optimized for *E. coli* and commercially synthesized (Thermo Fisher). The *E. coli* genes *gcvHLPT*, *lplA*, and *shmt*, as well as P. stutzeriptdh* (ptdh:D13E/M26I/V71I/E130 K/Q132R/Q137R/I150F/Q215L/R275Q/

L276Q/I313L/V315A/A319E/A325 V/E332N/C336D/E175A)^51^ were not codon optimized and commercially synthesized (Thermo Fisher). Clones were confirmed by DNA sequencing. All sequences used in this work can be found in Supporting InformationTables S6 and S7. Plasmids generated for this work can be found in Supporting InformationTable S5.

### Formate-to-Serine Pathway—Linear DNA

The genes *ftl*, *fch*, *mtdA*, ptdh*, *gcvHLPT*, *lplA*, *shmt* were amplified from their respective vectors using primers that bound ∼100 bp upstream from the promoter and downstream the terminator to protect the sequence from exonuclease degradation.^52^ Primers RW9/RW10 were used to amplify linear DNA from the P70a-based plasmids, while GH1/GH2 were used to amplify linear DNA from PTXTL-T3- and PTXTL-T7-based plasmids. The T3 RNA polymerase was amplified from pTXTL-P70a-T3rnap (Arbor Biosciences). The T7 RNA polymerase was amplified from pTXTL-P70a-T7rnap (Arbor Biosciences) using GH3/GH4, respectively.

### Formate-to-Serine Pathway—Tagged Genes for Western Blots

For the Western blot experiments, the Module 2 genes (*gcvHLPT* and *lplA*) were recloned into either pTXTL-P70a-deGFP, pTXTL-T7-deGFP, or pTXTL-T3-deGFP carrying a N-terminal His_6_-tag. For *gcvHLP* and *lplA*, the His_6_-tag was bracketed by GSS-His_6_-SSG, while for *gcvT*, the His_6_-tag was introduced after the starting methionine. A point mutation in *gcvP* (*gcvP*:A24V) is present in the His_6_-tag version of the gene, but not in the version used for chemical production. Clones were confirmed via DNA sequencing.

### GFP Expression from Plasmid and Linear DNA

Reactions were set up in a 96-well V bottom plate containing transcription–translation (TX/TL) mixture (75% vol), 5 nM of either pTXTL-P70a-deGFP (plasmid DNA) or P_70_-deGFP (linear DNA), and water to reach 5 μL. GFP was expressed for 16 h at 30 °C. GFP fluorescence (ex: 490 nm/em: 510 nm) was measured every 3 min using a TECAN plate reader.

### GFP Expression from Plasmid and Linear DNA after Dilution. GFP Expression with Dilution

Reactions were set up in a 96-well V bottom plate containing TX/TL mixture (75% vol), 5 nM of either pTXTL-P70a-deGFP (plasmid DNA) or P_70_-deGFP (linear DNA), and water to reach 5 μL. GFP was expressed for 1 h at 30 °C. Biocatalyst dilution step: biocatalysts were transferred to PCR tubes and either not diluted (1×) or diluted with water to 50 μL (10×), 200 μL (40×), 500 μL (100×), and 1000 μL (200×). Next, 5 μL samples from 1× to 200× diluted biocatalysts were transferred to a 96-well plate in order to monitor GFP expression. GFP was expressed for 3 h at 30 °C with fluorescence (ex: 490 nm/em: 510 nm) measured every 3 min using a TECAN plate reader.

### NADH Consumption of Plain CFE with Dilution

Reactions were set up in PCR tubes containing TX/TL mixture (75% vol) and water to reach 25 μL. Biocatalyst dilution step: 10× diluted plain CFE was obtained by moving the biocatalyst to a microcentrifuge tube and adding water to 250 μL. The reaction was then supplemented with 1 mM NADH and incubated for 4 h at 29 °C shaken at 0.0015*g*.

### Module 1: Formate to CH=THF

Reactions were set up in PCR tubes containing TX/TL mixture (75% vol), 5 nM each of p70a-*M. extorquens*_ftl and p70a-*M. extorquens*_fch, and water to reach 25 μL. Gene expression step: 1 h at 30 °C shaken at 2.5*g*. Biocatalyst dilution step: dilutions were obtained by moving the biocatalyst to a microcentrifuge tube and adding water to 250 μL (10×), 1 mL (40×), 2.5 mL (100×), and 5 mL (200×). Chemical synthesis step: 1 mM each of THF, formate, and ATP. Chemical synthesis took place over 3 h at 29 °C shaken at 0.0015*g*.

### Module 1: CH=THF to CH_2_–THF

Reactions were set up in PCR tubes containing TX/TL mixture (75% vol), 1 mM LiAC, and 5 nM each of p70a-*M. extorquens*_mtdA and p70a-*A. thaliana*_fdh*, and water to reach 25 μL. Gene expression step: 16 h at 30 °C shaken at 2.5*g*. Biocatalyst dilution step: 10× dilution was obtained by moving the biocatalyst to a microcentrifuge tube and adding water to 250 μL. Chemical synthesis step: 1 mM each of CH=THF, formate, and NADPH. Reactions were overlaid with argon, and sealed. Chemical synthesis took place over 3 h at 29 °C shaken at 0.0015*g*.

### NADPH Regeneration with fdh*

Reactions were set up in PCR tubes containing TX/TL mixture (75% vol), 5 nM of p70a-*A. thaliana*_fdh*, and water to reach 25 μL. Gene expression step: 16 h at 30 °C shaken at 2.5*g*. Biocatalyst dilution step: 10× dilution was obtained by moving the biocatalyst to a microcentrifuge tube and adding water to 250 μL. Chemical synthesis step: 1 mM NADP^+^. Chemical reaction took place over 3 h at 29 °C and was shaken at 0.0015*g*.

### Module 1: Formate to CH_2_–THF

Reactions were set up in PCR tubes containing TX/TL mixture (75% vol), 5 nM each of p70a-*M. extorquens*_ftl, p70a-*M. extorquens*_fch, p70a-*M. extorquens*_mtdA, and p70a-*A. thaliana*_fdh*, and water to reach 25 μL. Gene expression step: 1 or 16 h at 30 °C shaken at 2.5*g*. Biocatalyst dilution step: No 1× dilution was performed. 10× dilution was obtained by moving the biocatalyst to a microcentrifuge tube and adding water to 250 μL. Chemical synthesis step: stoichiometric concentrations of reactants and cofactors (1 mM each of THF, ATP, NADPH and 2 mM formate). Reactions were overlaid with argon, and sealed. Chemical synthesis took place over 3 h at 29 °C shaken at 0.0015*g*.

### Module 3: CH_2_–THF and Glycine to Serine

Reactions were set up in a 96-well plate using a Labcyte Echo 525. The reactions contained TX/TL (75% vol), 100 μM pyridoxal-5-phosphate (PLP), 5 nM p70a-*E. coli*_shmt, and water to reach 5 μL. Gene expression step: 16 h at 30 °C shaken at 2.5*g*. Biocatalyst dilution step: 10× dilution was obtained by moving the biocatalyst to a PCR tube and adding water to 50 μL. Chemical synthesis step: stoichiometric concentrations of reactants (1 mM each of CH_2_–THF and glycine). Chemical synthesis took place over 4 h at 29 °C shaken at 0.0015*g*.

### Module 1 + 3 + fdh*: Formate and Glycine to Serine Using fdh* for NADH Regeneration

Reactions were set up in a 96-well plate using a Labcyte Echo 525. The reactions contained 100 μM PLP and 5 nM each of p70a-*M. extorquens*_ftl, p70a-*M. extorquens*_fch, p70a-*M. extorquens*_mtdA, p70a-*A. thaliana*_fdh*, and p70a-*E. coli*_shmt. By hand, TX/TL (75% vol) and water were added to reach 5 μL. Gene expression step: 16 h at 30 °C shaken at 2.5*g*. Biocatalyst dilution step: 10× dilution was obtained by moving the biocatalyst to a PCR tube and adding water to 50 μL. Chemical synthesis step: stoichiometric concentrations of reactants and cofactors (1 mM each of THF, glycine, NADPH, ATP and 2 mM formate). Reactions were overlaid with argon, and sealed. Chemical synthesis took place over 4 h at 29 °C shaken at 0.0015*g*.

### Module 1 (Operon) + 3 + fdh*: Formate and Glycine to Serine Using fdh* for NADH Regeneration

Reactions were set up in a 96-well plate using a Labcyte Echo 525. The reactions contained 100 μM PLP and 5 nM each of p70a-*M. extorquens* ftl_fch_mtdA, p70a-*A. thaliana*_fdh*, and p70a-*E. coli*_shmt. By hand, TX/TL (75% vol) and water were added to reach 5 μL. Gene expression step: 16 h at 30 °C shaken at 2.5*g*. Biocatalyst dilution step: 10× dilution was obtained by moving the biocatalyst to a PCR tube and adding water to 50 μL. Chemical synthesis step: stoichiometric concentrations of reactants and cofactors (1 mM each of THF, glycine, NADPH, ATP, and 2 mM formate). Reactions were overlaid with argon, and sealed. Chemical synthesis took place over 4 h at 29 °C shaken at 0.0015*g*.

### Module 1 (Operon) + 3 + ptdh*: Formate and Glycine to Serine Using ptdh* for NADH Regeneration

Reactions were set up in a 96-well plate using a Labcyte Echo 525. The reactions cotained 100 μM PLP, and 5 nM each of p70a-*M. extorquens* mdtA_ftl_fch, p70a-*P.stutzeri*_ptdh*, and p70a-*E. coli*_shmt. By hand, TX/TL (75% vol) and water were added reach 5 μL. Gene expression step: 16 h at 30 °C shaken at 2.5*g*. Biocatalyst dilution step: 10× dilution was obtained by moving the biocatalyst to a PCR tube and adding water to 50 μL. Chemical synthesis step: stoichiometric concentrations of reactants and cofactors (1 mM each of formate, THF, glycine, NADPH, and ATP), and for NADPH regeneration 3 mM Na_2_HPO_3_. Reactions overlaid with argon, and sealed. Chemical synthesis took place over 4 h at 29 °C shaken at 0.0015*g*.

### Module 2 + 3 + ptdh*: CH_2_–THF, Ammonia, and Bicarbonate to Glycine and Serine

Reactions were set up in a 96-well plate using a Labcyte Echo 525. The reactions cotained 100 μM PLP and plasmids. By hand, TX/TL (75% vol), α-lipoic acid (100 μM final concentration) and water were added to reach 5 μL. For a plasmid DNA *gcvHLPT*/*lplA* 8:1:1:1:1 ratio: 40 nM of p70a-*E. coli*_gcvH and 5 nM each of P70a-*E. coli*_gcvL, p70a-*E. coli*_gcvP, p70a-*E. coli*_gcvT, p70a-*E. coli*_lplA, as well as 5 nM each of p70a-*E. coli*_shmt and p70a-*P. stutzeri*_ptdh* were dispensed. For a linear DNA *gcvHLPT*/*lplA* 96:3:1:4:4 ratio: 96 nM of P_T70_-*E. coli*_gcvH, 3 nM P_T70_-*E. coli*_gcvL, 1 nM P_T70_-*E. coli*_gcvP, 4 nM P_T70_-*E. coli*_gcvT, and 4 nM P_T70_-*E. coli*_lplA , as well as 3 nM each of P_T70_-*E. coli*_shmt and P_T70_-*E. coli*_ptdh* were dispensed. For a linear DNA *gcvHLPT*/*lplA* 192:2:1:4:2 ratio: 192 nM P_T70_-*E. coli*_gcvH or P_T3_-*E. coli*_gcvH, 2 nM P_T70_-*E. coli*_gcvL, 1 nM P_T70_-*E. coli*_gcvP, 4 nM P_T70_-*E. coli*_gcvT, 2 nM P_T70_-*E. coli*_lplA, as well as 3 nM each of P_T70_-*E. coli*_ptdh* and P_T70_-*E. coli*_shmt were added. For the reaction expressing P_T3_-*E. coli*_gcvH also contained 3 nM linear P_T70_-T3rnap. Gene expression step: 16 h at 30 °C shaken at 2.5*g*, followed by 2 h at 15 °C shaken at 1.5*g*. Biocatalyst dilution step: 10× dilution was obtained by moving the biocatalyst to a PCR tube and adding 0.1 M pH 8 Tris HCL to reach 50 μL. Chemical synthesis step: 20 mM DTT, 100 μM α-lipoic acid, 3 mM Na_2_HPO_3_, 2 mM CH_2_-THF and 1 mM each of NH_3_, NaHCO_3_, and NADH. The reactions were overlaid with argon and sealed. Chemical synthesis took place over 4 h at 29 °C shaken at 0.0015*g*.

### CFE-Based ptdh* Biocatalyst Substrate Preference

Reactions were set up in a 96-well plate using a Labcyte Echo 525. The reactions contained 5 nM linear P_T70_-ptdh*. By hand, TX/TL (75% vol) and water were added to reach 5 μL. Gene expression step: 16 h at 30 °C shaken at 2.5*g*. Biocatalyst dilution step: 10× diluted biocatalyst dx110x dilution was obtained by moving the biocatalyst to a PCR tube and diluted to 50 μL using water. Chemical synthesis step: 1 mM Na_2_HPO_3_, 1 mM NAD^+^, and 1 mM NADP^+^. Cofactor regeneration took place over 4 h at 29 °C shaken at 0.0015*g*.

### Module 1 + 2 + 3 + ptdh*: Formate, Ammonia, and Bicarbonate to Glycine and Serine

Reactions were set up in a 96-well plate using a Labcyte Echo 525. The reactions contained 100 μM PLP, 3 nM p70a-*M. extorquens* mdtA_ftl_fch, 192 nM P_T3_-*E. coli*_gcvH or P_T3_-*E. coli*_gcvH, 2 nM P_T70_-*E. coli*_gcvL, 1 nM P_T70_-*E. coli*_gcvP, 4 nM P_T70_-*E. coli*_gcvT, 2 nM P_T70_-*E. coli*_lplA, as well as 3 nM each of P_T70_-*E. coli*_ptdh*, P_T70_-*E. coli*_shmt, and P_T70_-T3RNA. By hand, TX/TL (75% vol), α-lipoic acid (100 μM final concentration), and water were added to reach 5 μL. Reactions with 2× *mtdA* contained 6 nM P_T70_-*E. coli*_*mtdA*. Reactions with 2× *mdtA* and 2× *shmt* contained 6 nM P_T70_-*E. coli*_*mtdA* and 6 nMP_T70_-*E. coli*_*shmt*. Gene expression step: 16 h at 30 °C shaken at 2.5*g*, followed by 2 h at 15 °C shaken at 1.5*g*. Biocatalyst dilution step: 10x diluted biocatalyst was moved to a PCR tube and brought up to 50 μL using 0.1 M pH 8 Tris HCL. Chemical synthesis step: 20 mM DTT, 100 μM α-lipoic acid, 2 mM each of THF, formate, NADPH, ATP, and 1 mM each of NH_3_, NaHCO_3_, NADH, and 3 mM Na_2_HPO_3_. Reactions with 0.1× THF contained 0.2 mM THF. The reactions were overlaid with argon and sealed. Chemical synthesis took place over 4 h at 29 °C shaken at 0.0015*g*.

### Module 2 Gene Expression-Western Blots

Reactions were set up in a 96-well plate using a Labcyte Echo 525. The reactions contained 100 μM PLP and various concentrations of plasmid (p70a-*E. coli*_GSS-His_6_-SSG-gcvH, p70a-*E. coli*_GSS-His_6_-SSG-His_6_-gcvL, p70a-*E. coli*_GSS-His_6_-SSG-gcvP:A24 V, p70a-*E. coli*_His_6_-gcvT, p70a-*E. coli* GSS-His_6_-SSG-lplA) or linear versions of the constructs. By hand, TX/TL (75% vol), α-lipoic acid (100 μM final concentration), and water were added to reach 5 μL. For P_T3_ and P_T7_ reactions also contained 3 nM P_T70_-T3RNA or P_T70_-T7RNA. Gene expression step: 16 h at 30 °C shaken at 2.5 g. Western blot: 2 μL samples of each reaction were heat denatured in the presence of NuPAGE LDS sample buffer, loaded in a 4–12% Bis-Tris gel and ran using an XCell SureLock Mini-Cell Electrophoresis System and NuPAGE MES SDS running buffer. The protein bands were transferred to nitrocellulose paper using an iBlot Dry Blotting System. Proteins were washed between steps with Tris buffer, blocked with a bovine serum albumin , and labeled with a mouse monoclonal anti-polyHistidine antibody followed by an anti-mouse IgG—alkaline phosphatase antibody. The blot was developed using a nitro-blue tetrazolium chloride and 5-bromo-4-chloro-3′-indolyphosphate *p*-toluidine salt color developing substrate system.

### Amino Acid Derivatization

For liquid chromatography/mass spectrometry (LC/MS) detection and quantification of serine and glycine, the amino acids were derivatized to their Fmoc protected versions using 9-fluorenylmethoxycarbonyl chloride (Fmoc-Cl).^53^ To the 10× diluted biocatalyst (50 μL), 50 μL of 5 % acetic acid in methanol and 2 μL of 50 mM Boc-Serine (internal standard) were added. The denatured reaction was centrifuged and 50 μL of the supernatant diluted to 500 μL using water. To 25 μL of the diluted supernatant, 100 μL of 3 mM Fmoc-Cl dissolved in acetone was added and the pH adjusted to 8.3 using saturated NaHCO_3_. The reaction was done at room temperature for 10 min. The Fmoc-derivatized amino acids were extracted using 500 μl of ethyl acetate (3×), dried under vacuum, and resuspended in 200 μL of methanol for analysis.

### Metabolite Quantification

All other metabolites were detected and quantified via LC/MS without derivatization. The proteins in the 10× diluted biocatalyst were denatured by adding 5% acetic acid in methanol spiked with 4 mM catechol (internal standard). The denatured reactions were centrifuged at 16,000*g* for 15 min and the supernatant directly analyzed. LC/MS conditions: THF, CH=THF, CH_2_–THF, NAD^+^, NADPH, NADP^+^, NADH were quantified using an Agilent 1100/1260 HPLC equipped with an Agilent 6120 Single Quadrupole MS, using a Poroshell 120 SB-C_18_ 3.0 mm × 50 mm × 2.7 μm column and an electrospray ion source. Column temperature was kept constant at 28 °C. The LC method was based on Chen et al.^54^ LC conditions: Solvent A—water with 3% methanol, 10 mM tributylamine, and 15 mM acetic acid, Solvent B—methanol. Gradient: 0 min, 0% B; 2.5 min, 0% B; 5 min, 50% B; 14 min, 95% B; 15 min, 0% B; and 20 min, 0% B. MS acquisition: Selective ion monitoring in negative ion mode was used to detect and quantify THF (*m*/*z* 444), CH=THF (*m*/*z* 454), and CH_2_–THF (*m*/*z* 456). Positive ion mode was used to detect and quantify NADH (*m*/*z* = 666), NAD^+^ (*m*/*z* = 664), NADPH (*m*/*z* = 746), and NADP^+^ (*m*/*z* = 104). Commercial THF, CH=THF, CH_2_–THF, NADH, NAD^+^, NADPH, and NADP^+^ were used to determine retention times and generate standard curves for chemical quantification. The Fmoc-derivatized amino acids were quantified using an Agilent 1260 Infinity II HPLC system equipped with an Agilent Q-TOF 6530 detector, using a Poroshell 120 SB-C_18_ 3.0 × 50 × 2.7 μm column. LC conditions: Solvent A—water with 0.1% formic acid, Solvent B—methanol with 0.1% formic acid. Gradient: 0 min, 0% B; 2.5 min, 0% B; 5 min, 50% B; 15 min, 100% B; 15 min, 0% B; 20 min, 0% B. MS acquisition: Extracted ion chromatogram in positive ion mode was used to detect and quantify Fmoc-Serine (*m*/*z* 328.11) and Fmoc-Glycine (*m*/*z* 298.11). Fmoc derivatized commercial glycine and serine were used to determine retention times and generate standard curves for chemical quantification.

## Data Availability

All the data generated or analyzed during this study are included in the published article and its Supporting Information files.

## Supporting Information Available

Reagents; solvents; kits; plasmids; primers; promoters; sequences of genes evaluated; LC/MS traces of commercial THF, 5,10-methenyltetrahydrofolate (CH=THF), 5,10 methyleneltetrahydrofolate (CH_2_–THF), NADPH, NADP^+^, NADPH, NAD^+^ in plain CFE; LC/MS traces of commercial Fmoc-Serine and Fmoc-glycine in plain CFE; standard curves using commercial THF, 5,10-methenyltetrahydrofolate (CH=THF), and 5,10 methyleneltetrahydrofolate (CH_2_–THF); standard curves using commercial NADH, NAD^+^, NADPH, and NADP^+^; standard curves of Fmoc-Serine and Fmoc-Glycine; and complete Western blots (PDF)

## Notes

^∇^ Lead contact.
