# Supplementary material for: Carbon Negative Synthesis of Amino Acids Using a Cell-Free-Based Biocatalyst
Source: ACS Synth Biol. 2024 Nov 21;13(12):3961–75. doi: 10.1021/acssynbio.4c00359 (PMC11669175; doi:10.1021/acssynbio.4c00359)
Supplement: Supplementary file 1 — sb4c00359_si_001.pdf [file sb4c00359_si_001.pdf]

Carbon negative synthesis of amino acids using a cell-free-based biocatalyst

Shaafique Chowdhury[1], Ray Westenberg[1,2], Kimberly Wennerholm[3], Ryan A. L. Cardiff[4], Alexander S. Beliaev[5,6], Vincent Noireaux[7], James M. Carothers[4,8]*, Pamela Peralta-Yahya[1,2,3,9]*

[1] School of Chemical & Biomolecular Engineering, Georgia Institute of Technology, Atlanta, GA 30332

[2] Bioengineering Program, Georgia Institute of Technology, Atlanta, GA 30332

[3] School of Chemistry and Biochemistry, Georgia Institute of Technology, Atlanta, GA 30332

[4] Molecular Engineering & Sciences Institute and Center for Synthetic Biology, University of Washington, Seattle, WA 98195, USA

[5] Environmental Molecular Sciences Division, Pacific Northwest National Laboratory, Richland, WA, 99354, USA

[6] Centre for Agriculture and the Bioeconomy, School of Biological and Environmental Sciences, Queensland University of Technology, Gardens Point Campus, PO Box 2434, Brisbane 4001, Queensland, Australia

[7] School of Physics and Astronomy, University of Minnesota, Minneapolis, MN 55455, USA

[8] Department of Chemical Engineering, University of Washington, Seattle, WA 98195, USA

[9] Lead contact

* Correspondence to:

E-mail: jcaroth@uw.edu

E-mail: pperalta-yahya@chemistry.gatech.edu

Supplementary Information

Table of Contents

	Page
**Table S1.** Table of Reagents	**SI2**
**Table S2.** Table of Solvents	**SI3**
**Table S3.** Table of Kits	**SI3**
**Table S4.** Table of primers	**SI3**
**Table S5.** Table of plasmids	**SI4**
**Table S6.** Table of promoters	**SI5**
**Table S7.** Sequences of genes evaluated	**SI5**
**Figure S1.** LC/MS traces of commercial tetrahydrofolate (THF), 5,10-methenyltetrahydrofolate (CH=THF), 5,10 methyleneltetrahydrofolate (CH_2-THF), NADPH, $NADP^+$, NADPH, NAD^+ in plain cell-free expression (CFE).	**SI10**
**Figure S2.** LC/MS traces of commercial Fmoc-Serine and Fmoc-glycine in plain cell-free expression (CFE).	**SI11**
**Figure S3.** Standard curves using commercial tetrahydrofolate (THF), 5,10-methenyltetrahydrofolate (CH=THF) and 5,10 methyleneltetrahydrofolate (CH_2-THF).	**SI12**
**Figure S4.** Standard curves using commercial NADH, NAD^+, NADPH and $NADP^+$.	**SI13**
**Figure S5.** Standard curves of Fmoc-Serine and Fmoc-Glycine.	**SI14**
**Figure S6.** Complete Western blots Figure 4D	**SI15**
**Figure S7.** Complete Western blots Figure 4E	**SI15**
**References**	**SI15**

Table S1: Table of Reagents.

Reagents	Vendor	Catalog #
1,4-dithiothreitol (DTT)	Sigma	12/3/3483
25% ammonia in water	Millipore Sigma	1.05422
5,10 methylene tetrahydrofolate	Cayman Chemicals	33967
5,10-methenyl tetrahydrofolate	Cayman Chemicals	31333
ATP	Millipore Sigma	A6419
catechol	Millipore Sigma	PHL823720
Fmoc Chloride	Oakwood Chemical	22072
Formic acid	Fischer scientific	A117-50
Glycine	Millipore Sigma	G7126
NADH	Cayman Chemicals	16078
NADPH	Cayman Chemicals	9000743
Pyridoxal-5-phosphate	TCI chemicals	C0377
Serine	Millipore Sigma	S4500
Sodium bicarbonate	Millipore Sigma	S5761
Sodium dihydrogen phosphate	Millipore Sigma	1.0637
Tetrahydrofolate	Cayman Chemicals	18263
α-lipoic acid	Millipore Sigma	1368301
Na_2HPO_3	Millipore Sigma	04283
NuPAGE™ 4 to 12%, Bis-Tris, 1.0–1.5 mm, Mini Protein Gels	Invitrogen	NP0329BOX
NuPAGE™ LDS Sample Buffer (4X)	Invitrogen	NP0007
NuPAGE™ MES SDS Running Buffer (20X)	Invitrogen	NP0002
PageRuler prestained protein ladder	Thermo Scientific	26616
N-terminal HIS-tagged Green Fluorescent Protein	Millipore Sigma	14-392
iBlot™ Transfer Stack, nitrocellulose, mini	Invitrogen	IB301002
nitro-blue tetrazolium chloride		
5-bromo-4-chloro-3'-indolyphosphate p-toluidine salt		
Monoclonal Anti-poly istidine antibody produced in mouse	Millipore Sigma	H1029
Anti-Mouse IgG (whole molecule)−Alkaline Phosphatase antibody produced in goat	Millipore Sigma	A3688

Table S2: Table of Solvents

Reagents	Vendor	Catalog #
Acetic acid	EMD Millipore	101830
Methanol	Fischer Scientific	A452-4
Tributylamine	Sigma	90780
Ethyl Acetate	Sigma	319902
Acetone	Fischer Scientific	326801000

Table S3: Table of Kits

Kit	Vendor	Catalog #
myTXTL Sigma 70 master mix	Arbor Biosciences	507096
CFE linear DNA kit	Arbor Biosciences	508096
XCell SureLock™ Mini-Cell	Invitrogen	EI0001

Table S4: Table of primers

Primer Name	Sequence
SC12	GCGGTGATAATGGTTGCAG
JS4	ACTGGGTTGAAGGCTCTCAA
RW9	GACTATCGCACCATCAGC
RW10	CTGTCCTACGAGTTGCATG
GH1	GTGATGTCGGCGATATAGGC
GH2	CTGTCCGACCGCTTTG
GH3	CGCCTGATGCGTGAAC
GH4	GTAGCACCTGAAGTCAGCC

Table S5: Table of plasmids

Strain number	Plasmid name	Description	Source
PPY2510	pTXTL-P70a-deGFP		Arbor Biosciences #502056
PPY2526	pTXTL-T3P-deGFP		Arbor Biosciences #502104
PPY2525	pTXTL-T7-deGFP		Arbor biosciences #503002
PPY2528	pTXTL-P70a-T3rnap		Arbor biosciences #502081
PPY2529	pTXTL-P70a-T7rnap		Arbor biosciences #502072
PPY2536	pRW20	p70a-*M.extorquens*_fch	This Study
PPY2610	pSC38	p70a-*M.extorquens*_ftl	This Study
PPY2611	pSC39	p70a-*M.extorquens*_mtdA	This Study
PPY2537	pRW21	p70a-*E. coli*_gcvH	This Study
PPY2551	pRW35	p70a-*E.coli*_gcvL	This Study
PPY2542	pRW26	p70a-*E.coli*_gcvP	This Study
PPY2619	pKW24	p70a-*E.coli*_gcvT	This Study
PPY2538	pRW22	p70a-*E.coli*_lplA	This Study
PPY2539	pRW23	p70a-*E.coli*_shmt	This Study
PPY2552	pRW36	p70a-*M.extorquens* mdtA_ftl_fch	This Study
PPY2540	pRW24	p70a-*A.thaliana*_fdh*	This Study
PPY2541	pRW25	p70a-*P.stutzeri*_ptdh*	This Study
PPY2544	pRW28	p70a- *E.coli*_GSS-His_6-SSG-gcvH	This Study
PPY2546	pRW30	p70a-*E.coli*_GSS-His_6-SSG-His_6-gcvL	This Study
PPY2591	pKW21	p70a-*E.coli*_ GSS-His_6-SSG-gcvP:A24V	This Study
PPY2550	pRW34	p70a-*E.coli*_His_6-gcvT	This Study
PPY2545	pRW29	p70a-*E.coli* GSS-His_6-SSG-lplA	This Study
PPY2580	pRW59	pT3-*E. coli*_His_6-gcvH	This Study
PPY2980	pKW30	pT7-*E. coli*_His_6-gcvH	This Study
PPY2598	pSC31	pT3-*E. coli*_His_6-lplA	This Study
PPY2602	pSC35	pT7-*E. coli*_His_6-lplA	This Study
PPY2575	pKW05	pT3-*E. coli*_gcvH	This Study

Table S6: Table of promoters and terminator

Promoter	Sequence
P_{T70}	TGAGCTAACACCGTGCGTGTTGACAATTTTACCTCTGGCGGTGATAATG<br>GTTGCA**<u>GCTAGC</u>**
T_{500} [PT70 TERMINATOR]	**<u>CTCGAG</u>**CAAAGCCCGCCGAAAGGCGGGCAAAGCCCGCCGAAAGGCGG<br>GCTTTTCTGT
P_{T3}	ATTAACCCTCACTAAAGGGAGACCACAACGGTTTCCCTCTAGAAATAATT<br>TTGTTTAACTTTAAGAAGGAGATATA**<u>CCATGG</u>**
P_{T7}	TAATACGACTCACTATAGGGAGACCACAACGGTTTCCCTCTAGAAATAAT<br>TTTGTTTAACTTTAAGAAGGAGATATA**<u>CCATGG</u>**
T_{T7} [PT7 AND PT3 TERMINATOR]	**<u>CTCGAG</u>**CCTTAGGAGATCCGGCTGCTAACAAAGCCCGAAAGGAAGCTG<br>AGTTGGCTGCTGCCACCGCTGAGCAATAACTAGCATAACCCCTTGGGG<br>CCTCTAAACGGGTCTTGAGGGGTTTTTTGCTGAAAGGAGGAACTATA

Nhe, NcoI, XhoI sites **<u>bold and underlined</u>**

Table S7: Sequences of genes evaluated

Origin	Gene	Enzyme	Notes	Sequences used
Methylobacterium extorquens	*ftl*	formate-THF ligase	Q83WS0 (optimized)	atgccgagcgatattgaaattgcacgcgctgctactctgaaaccgattgcgcaagttgcgga<br>gaaactgggtattccggacgaggctcttcataattatggcaaacatatcgctaaaatcgacca<br>tgactttattgcttctcttgagggtaaaccagagggcaaacttgttctggttactgctatttcgccg<br>actccagctggcgagggcaaaactactactactgttggtctgggcgatgctctcaaccgcatt<br>ggcaaacgtgctgttatgtgtctgcgcgagccctctctcggcccctgttttggcatgaaaggcg<br>gcgctgctggtggcggcaaagctcaggttgttccgatggagcagattaatctgcacttcaccg<br>gcgattttcacgctattacttctgctcactctctcgctgctgctctgattgataaccatatttattggg<br>ctaacgaactgaatattgacgttcgccgcattcattggcgccgcgttgttgatatgaacgatcg<br>ggctctgcgcgctattaatcagtctctcggcggcgttgctaatggctttccgcgcgaggatgggt<br>ttgacattactgttgcttctgaggttatggctgtgttttgcctcgccaagaatctggctgatcttgag<br>gagcggctcggccgcattgttattgcagaaactcgcgatcgcaaaccggttactctggctgat<br>gttaaagctactggcgctatgactgttctgctcaaggatgctcttcagccgaatctcgtgcagac<br>tctggagggcaacccggctctgattcacggcggcccgtttgctaacattgctcatggctgtaac<br>tcggttattgctactcgcactggcctgcggctcgctgactatactgttactgaggctggctttggc<br>gctgatctcggcgctgagaaattcattgatattaaatgtcgccagactggcctcaagccctctg<br>ctgttgttattgttgctacgattcgcgctctcaaaatgcatggcggcgttaacaagaaagatctc<br>caggctgagaatctggatgcgctggagaaaggttttgcaaatcttgagcgccatgttcacaat<br>gttcgctcttttggcctgccggttgttgttggtgttaaccacttctttcaggatactgatgctgagcat<br>gttcggttgaaagaactgtgccgcgatcggcttcaggttgaggctattacttgtaagcattgggc<br>tgagggcggcgcaggcgcagaagcactggcacaggcagttgttaaactggctgaaggcg<br>agcagaaaccgctgacttttgcatatgagaccgaaactaagattactgacaagattaaggca<br>attgctactaaactgtatggtgctgctgatattcagattgagtctaaagccgccactaagctcgc<br>tggcttcgagaaagatggctatggtaagctgccggtctgtatggccaagactcaatattcatttt<br>ctactgatccgactcttatgggcgctccctctggtcatctggtttctgtgcgcgatgttcgcctctct<br>gctggcgctggcttcgttgttgttatttgtggtgagattatgaccatgccgggtctgccgaaggttc<br>cagcagcagatactattcgcctcgatgctaacggtcagattgatgggctgttctag

	fch	methenyl-THF cyclohydrolase	Q49135 (optimized)	atggctggcaatgagactattgaaacattcttggacggcctggcatcatctgctccgactcccggcggcggcggtgcagcagcaatttctggcgcaatgggcgcagcacttgtttctatggtttgcaatcttactattggcaagaagaaatatgttgaggttgaggcagacttaaaacaggttctggagaaatctgaaggcctgcgccgcactctcactggcatgattgcagacgacgttgaagcctttgacgcagttatgggcgcttatgggctgccgaagaatactgacgaagagaaagcagcacgcgcagcaaagattcaagaggcactcaaaactgcaactgacgttccgctcgcatgttgtcgcgtttgtcgcgaggttattgatctggcagagattgttgcagagaaaggcaatctcaatgttatttctgatgcaggcgttgcagtgctctctgcttatgcaggtctgcgctctgctgcacttaatgtctatgtaaatgcaaaaggcctcgacgaccgcgcatttgcagaggagcggcttaaagagctggagggcctactggctgaggcaggtgcactcaatgagcgaatttatgagactgttaaatctaaagtgaattga
	mtdA	methylene THF dehydrogase	P55818 (optimized)	atgtctaagaaactgctctttcagtttgacactgatgcaactccgtctgtatttgacgttgttggctatgacggcggtgcagaccatattactggctatggcaatgttactcccgacaatgttggcgcatatgttgacggcactatttatactcgtggaggcaaagagaaacagtctacagcaatctttgttggcggcggcgacatggcagcaggcgagcgggtatttgaggcagtaaagaagcgtttctttggcccgtttcgcgtttcttgtatgctggattctaatggctctaatactactgcagcagcaggcgttgcactcgttgttaaagcagcaggcggctctgttaaaggcaagaaagcagttgttctcgcaggtactggtccggttggtatgcgctctgcagctctgttagccggcgagggcgcagaggttgttctgtgtgggcgcaaactcgacaaagcacaggcagcagcagattctgttaataaacgcttcaaagttaatgttactgcagcagagactgcagacgacgcatctcgcgcagaggccgtgaaaggcgcacattttgtctttactgcaggtgcaattggccttgaactgctgccgcaggcagcatggcagaatgagtcttctattgaaattgtggccgattataatgcacagccgccgctcggcattggcgggattgatgcaactgacaaaggcaaagaatatggcggaaaacgcgcatttggtgcgctcggcattggcggcttgaaactcaaactgcatcgcgcatgtattgcaaaactgtttgagtcttctgaaggtgtatttgatgcagaggagatttataaactggcaaaagaaatggcatga
Escherichia coli	*gcvH*	glycine cleavage system (gcv) H protein	P0A6T9	atgagcaacgtaccagcagaactgaaatacagcaaagaacacgaatggctgcgtaaagaagccgacggcacttacaccgttggtattaccgaacatgctcaggagctgttaggcgatatggtgtttgttgacctgccggaagtgggcgcaacggttagcgcgggcgatgactgcgcggttgccgaatcggtaaaagcggcgtcagacatttatgcgccagtaagcggtgaaatcgtggcggtaaacgacgcactgagcgattccccggaactggtgaacagcgaaccgtatgcaggcggctggatctttaaaatcaaagccagcgatgaaagcgaactggaatcactgctggatgcgaccgcatacgaagcattgttagaagacgagtaa
	gcvL	gcv L protein	P0A9P0	atgagtactgaaatcaaaactcaggtcgtggtacttggggcaggccccgcaggttactccgctgccttccgttgcgctgatttaggtctggaaaccgtaatcgtagaacgttacaacacccttggcggtgtttgcctgaacgtcggctgtatcccttctaaagcactgctgcacgtagcaaaagttatcgaagaagccaaagcgctggctgaacacggtatcgtcttcggcgaaccgaaaaccgatatcgacaagattcgtacctggaaagagaaagtgatcaatcagctgaccggtggtctggctggtatggcgaaaggccgcaaagtcaaagtggtcaacggtctgggtaaattcaccggggctaacaccctggaagttgaaggtgagaacggcaaaaccgtgatcaacttcgacaacgcgatcattgcagcgggttctcgcccgatccaactgccgtttattccgcatgaagatccgcgtatctgggactccactgacgcgctggaactgaaagaagtaccagaacgcctgctggtaatgggtggcggtatcatcggtctggaaatgggcaccgtttaccacgcgctgggttcacagattgacgtggttgaaatgttcgaccaggttatcccggcagctgacaaagacatcgttaaagtcttcaccaagcgtatcagcaagaaattcaacctgatgctggaaaccaaagttaccgccgttgaagcgaaagaagacggcatttatgtgacgatggaaggcaaaaaagcacccgctgaaccgcagcgttacgacgccgtgctggtagcgattggtcgtgtgccgaacggtaaaaacctcgacgcaggcaaagcaggcgtggaagttgacgaccgtggtttcatccgcgttgacaaacagctgcgtaccaacgtaccgcacatctttgctatcggcgatatcgtcggtcaaccgatgctggcacacaaaggtgttcacgaaggtcacgttgccgctgaagttatcgccggtaagaaacactacttcgatccgaaagttatcccgtccatcgcctataccgaaccagaagttgcatgggtgggtctgactgagaaagaagcgaaagagaaaggcatcagctatgaaaccgccaccttcccgtgggctgcttctggtcgtgctatcgcttccgactgcgcagacggtatgaccaagctgattttcgacaaagaatctcaccgtgtgatcggtggtgcgattgtcggtactaacggcggcgagctgctgggtgaaatcggcctggcaatcgaaatgggttgtgatgctgaagacatcgcactgaccatccacgcgcacccgactctgcacgagtctgtgggcctggcggcagaagtgttcgaaggtagcattaccgacctgccgaacccgaaagcgaagaagaagtaa

gcvP	gcvP protein	P33195	atgacacagacgttaagccagcttgaaaacagcggcgcttttattgaacgccatatcggacc<br>ggacgccgcgcaacagcaagaaatgctgaatgccgttggtgcacaatcgttaaacgcgct<br>gaccggccagattgtgccgaaagatattcaacttgcgacaccaccgcaggttggcgcaccg<br>gcgaccgaatacgccgcactggcagaactcaaggctattgccagtcgcaataaacgcttca<br>cgtcttacatcggcatgggttacaccgccgtgcagctaccgccggttatcctgcgtaacatgct<br>ggaaaatccgggctggtataccgcgtacactccgtatcaacctgaagtctcccagggccgc<br>cttgaagcactgctcaacttccagcaggtaacgctggatttgactggactggatatggcctctg<br>cttctcttctggacgaggccaccgctgccgccgaagcaatggcgatggcgaaacgcgtcag<br>caaactgaaaaatgccaaccgcttcttcgtggcttccgatgtgcatccgcaaacgctggatgt<br>ggtccgtactcgtgccgaaacctttggttttgaagtgattgtcgatgacgcgcaaaaagtgctc<br>gaccatcaggacgtcttcggcgtgctgttacagcaggtaggcactaccggtgaaattcacga<br>ctacactgcgcttattagcgaactgaaatcacgcaaaattgtggtcagcgttgccgccgatatt<br>atggcgctggtgctgttaactgcgccgggtaaacagggcgcggatattgtttttggttcggcgc<br>aacgcttcggcgtgccgatgggctacggtggcccacacgcggcattctttgcggcgaaagat<br>gaatacaaacgctcaatgccgggccgtattatcggtgtatcgaaagatgcagctggcaatac<br>cgcgctgcgcatggcgatgcagactcgcgagcaacatatccgccgtgagaaagcgaactc<br>caacatttgtacttcccaggtactgctggcaaacatcgccagcctgtatgccgtttatcacggc<br>ccggttggcctgaaacgtatcgctaaccgcattcaccgtctgaccgatatcctggcggcggg<br>cctgcaacaaaaaggtctgaaactgcgccatgcgcactatttcgacaccttgtgtgtggaagt<br>ggccgacaaagcgggcgtactgacgcgtgccgaagcggctgaaatcaacctgcgtagcg<br>atattctgaacgcggttgggatcacccttgatgaaacaaccacgcgtgaaaacgtaatgcag<br>cttttcaacgtgctgctgggcgataaccacggcctggacatcgacacgctggacaaagacgt<br>ggctcacgacagccgctctatccagcctgcgatgctgcgcgacgacgaaatcctcacccat<br>ccggtgtttaatcgctaccacagcgaaaccgaaatgatgcgctatatgcactcgctggagcgt<br>aaagatctggcgctgaatcaggcgatgatcccgctgggttcctgcaccatgaaactgaacgc<br>cgccgccgagatgatcccaatcacctggccggaatttgccgaactgcacccgttctgcccgc<br>cggagcaggccgaaggttatcagcagatgattgcgcagctggctgactggctggtgaaact<br>gaccggttacgacgccgtttgtatgcagccgaactctggcgcacagggcgaatacgcgggc<br>ctgctggcgattcgtcattatcatgaaagccgcaacgaagggcatcgcgatatctgcctgatc<br>ccggcttctgcgcacggaactaaccccgcttctgcacatatggcaggaatgcaggtggtggtt<br>gtggcgtgtgataaaaacggcaacatcgatctgactgatctgcgcgcgaaagcggaacag<br>gcgggcgataacctctcctgtatcatggtgacttatccttctacccacggcgtgtatgaagaaa<br>cgatccgtgaagtgtgtgaagtcgtgcatcagttcggcggtcaggtttaccttgatggcgcgaa<br>catgaacgcccaggttggcatcacctcgccgggctttattggtgcggacgtttcacaccttaac<br>ctacataaaactttctgcattccgcacggcggtggtggtccgggtatgggaccgatcggcgtg<br>aaagcgcatttggcaccgtttgtaccgggtcatagcgtggtgcaaatcgaaggcatgttaacc<br>cgtcagggcgcggtttctgcggcaccgttcggtagcgcctctatcctgccaatcagctggatgt<br>acatccgcatgatgggcgcagaagggctgaaaaaagcaagccaggtggcaatcctcaac<br>gccaactatattgccagccgcctgcaggatgccttcccggtgctgtataccggtcgcgacggt<br>cgcgtggcgcacgaatgtattctcgatattcgcccgctgaaagaagaaaccggcatcagcg<br>agctggatattgccaagcgcctgatcgactacggtttccacgcgccgacgatgtcgttcccgg<br>tggcgggtacgctgatggttgaaccgactgaatctgaaagcaaagtggaactggatcgcttt<br>atcgacgcgatgctggctatccgcgcagaaattgaccaggtgaaagccggtgtctggccgct<br>ggaagataacccgctggtgaacgcgccgcacattcagagcgaactggtcgccgagtgggc<br>gcatccgtacagccgtgaagttgcggtattcccggcaggtgtggcagacaaatactggccg<br>acagtgaaacgtctggatgatgtttacggcgaccgtaacctgttctgctcctgcgtaccgatta<br>gcgaataccagtaa

Escherichia coli	*gcvT*	glycine cleavage system T protein	P27248	atggcacaacagactcctttgtacgaacaacacacgctttgcggcgctcgcatggtggatttc<br>cacggctggatgatgccgctgcattacggttcgcaaatcgacgaacatcatgcggtacgtac<br>cgatgccggaatgtttgatgtgtcacatatgaccatcgtcgatcttcgcggcagccgcacccg<br>ggagtttctgcgttatctgctggcgaacgatgtggcgaagctcaccaaaagcggcaaagcc<br>ctttactcggggatgttgaatgcctctggcggtgtgatagatgacctcatcgtctactactttactg<br>aagatttcttccgcctcgttgttaactccgccacccgcgaaaaagacctctcctggattaccca<br>acacgctgaacctttcggcatcgaaattaccgttcgtgatgacctttccatgattgccgtgcaag<br>ggccgaatgcgcaggcaaaagctgccacactgtttaatgacgcccagcgtcaggcggtgg<br>aagggatgaaaccgttctttggcgtgcaggcgggcgatctgtttattgccaccactggttatac<br>cggtgaagcgggctatgaaattgcgctgcccaatgaaaaagcggccgatttctggcgtgcg<br>ctggtggaagcgggtgttaagccatgtggcttgggcgcgcgtgacacgctgcgtctggaagc<br>gggcatgaatctttatggtcaggagatggacgaaaccatctctcctttagccgccaacatggg<br>ctggaccatcgcctgggaaccggcagatcgtgactttatcggtcgtgaagccctggaagtgc<br>agcgtgagcatggtacagaaaaactggttggtctggtgatgaccgaaaaaggcgtgctgcg<br>taatgaactgccggtacgctttaccgatgcgcagggcaaccagcatgaaggcattatcacca<br>gcggtactttctccccgacgctgggttacagcattgcgctggcgcgcgtgccggaaggtattg<br>gcgaaacggcgattgtgcaaattcgcaaccgtgaaatgccggttaaagtgacaaaacctgtt<br>tttgtgcgtaacggcaaagccgtcgcgtaa
	lpla	lipoate-protein ligase	P32099	atgtccacattacgcctgctcatctctgactcttacgacccgtggtttaacctggcggtggaaga<br>gtgtatttttcgccaaatgcccgccacgcagcgcgttctgtttctctggcgcaatgccgacacg<br>gtagtaattggtcgcgcgcagaacccgtggaaagagtgtaatacccggcggatggaagaa<br>gataacgtccgcctggcgcgacgcagtagcggtggcggtgcagtgttccacgatctcggca<br>atacctgctttacctttatggctggcaagccggagtacgataaaactatctccacgtcgattgtg<br>ctcaatgcgctgaacgcgctcggcgtcagcgccgaagcgtccggacgtaacgatctggtgg<br>tgaaaaccgtcgaaggcgaccgcaaagtctcaggctcggcctatcgcgaaaccaaagatc<br>gcggcttccaccacggcaccttgctactcaatgccgacctcagccgcctggcaaactatctc<br>aatccggataaaaagaaactggcggcgaaaggcattacgtcggtacgttcccgcgtgacc<br>aacctcaccgagctgttgccggggatcacccatgagcaggtttgcgaggccataaccgagg<br>cctttttcgcccattatggcgagcgcgtggaagcggaaatcatctccccgaacaaaacgcca<br>gacttgccaaacttcgccgaaacctttgcccgccagagtagctgggaatggaacttcggtca<br>ggctccggcattctcgcatctgctggatgaacgctttacctggggcggcgtggaactgcatttc<br>gacgttgaaaaaggccatatcacccgcgcacaggtgtttaccgacagcctcaacccagcg<br>ccgctggaagccctcgccggacgactgcaaggctgcctgtaccgcgcagatatgctgcaac<br>aggagtgcgaagcgctgttggttgacttcccggaacaggaaaaagagctacgggagttatc<br>ggcatggatggcgggggctgtaaggtag
Escherichia coli	*shmt*	Serine hydroxy methyltra nsferase	P0A825	atgttaaagcgtgaaatgaacattgccgattatgatgccgaactgtggcaggctatggagca<br>ggaaaaagtacgtcaggaagagcacatcgaactgatcgcctccgaaaactacaccagcc<br>cgcgcgtaatgcaggcgcagggttctcagctgaccaacaaatatgctgaaggttatccggg<br>caaacgctactacggcggttgcgagtatgttgatatcgttgaacaactggcgatcgatcgtgc<br>gaaagaactgttcggcgctgactacgctaacgtccagccgcactccggctcccaggctaact<br>ttgcggtctacaccgcgctgctggaaccaggtgataccgttctgggtatgaacctggcgcatg<br>gcggtcacctgactcacggttctccggttaacttctccggtaaactgtacaacatcgttccttac<br>ggtatcgatgctaccggtcatatcgactacgccgatctggaaaaacaagccaaagaacac<br>aagccgaaaatgattatcggtggtttctctgcatattccggcgtggtggactgggcgaaaatgc<br>gtgaaatcgctgacagcatcggtgcttacctgttcgttgatatggcgcacgttgcgggcctggtt<br>gctgctggcgtctacccgaacccggttcctcatgctcacgttgttactaccaccactcacaaaa<br>ccctggcgggtccgcgcggcggcctgatcctggcgaaaggtggtagcgaagagctgtaca<br>aaaaactgaactctgccgttttccctggtggtcagggcggtccgttgatgcacgtaatcgccgg<br>taaagcggttgctctgaaagaagcgatggagcctgagttcaaaacttaccagcagcaggtc<br>gctaaaaacgctaaagcgatggtagaagtgttcctcgagcgcggctacaaagtggtttccgg<br>cggcactgataaccacctgttcctggttgatctggttgataaaaacctgaccggtaaagaagc<br>agacgccgctctgggccgtgctaacatcaccgtcaacaaaaacagcgtaccgaacgatcc<br>gaagagcccgtttgtgacctccggtattcgtgtaggtactccggcgattacccgtcgcggcttta<br>aagaagccgaagcgaaagaactggctggctggatgtgtgacgtgctggacagcatcaatg<br>atgaagccgttatcgagcgcatcaaaggtaaagttctcgacatctgcgcacgttacccggttt<br>acgcataa

Pseudomonas stutzeri	*ptdh**	Phosphite dehydrogenase mutant	17X-PTDH from Ref. 1 Mutations: D13E, M26I, V71I, E130K, Q132R, Q137R, I150F, Q215L, R275Q, L276Q, I313L, V315A, A319E, A325V, E332N, C336D. E175A	atgctgccgaaactcgttataactcaccgagtacacgaagagatcctgcaactgctggcgcc<br>acattgcgagctgataaccaaccagaccgacagcacgctgacgcgcgaggaaattctgcg<br>ccgctgtcgcgatgctcaggcgatgatggcgttcatgcccgatcgggtcgatgcagactttctt<br>caagcctgccctgagctgcgtgtaatcggctgcgcgctcaagggcttcgacaatttcgatgtg<br>gacgcctgtactgcccgcggggtctggctgaccttcgtgcctgatctgttgacggtcccgactg<br>ccgagctggcgatcggactggcggtggggctggggaggcatctgagggcagcagatgcgt<br>tcgtccgctctggcaagttccggggctggcaaccacggttctacggcacggggctggataac<br>gctacggtcggcttccttggcatgggcgccatcggactggccatggctgatcgcttgcaggga<br>tggggcgcgaccctgcagtaccacgcggcgaaggctctggatacacaaaccgagcaacg<br>gctcggcctgcgccaggtggcgtgcagcgaactcttcgccagctcggacttcatcctgctggc<br>gcttcccttgaatgccgataccctgcatctggtcaacgccgagctgcttgccctcgtacggccg<br>ggcgctctgcttgtaaacccctgtcgtggctcggtagtggatgaagccgccgtgctcgcggcg<br>cttgagcgaggccagctaggagggtatgcggcggatgtattcgaaatggaagactgggctc<br>gcgcggacaggccacagcagatcgatcctgcgctgctcgcgcatccgaatacgctgttcact<br>ccgcacatagggtcggcagtgcgcgcggtgcgactggagattgaacgttgtgcagcgcag<br>aacatcctccaggcattggcaggtgagcgcccaatcaacgctgtgaaccgtctgcccaagg<br>ccgagcctgccgcatgttga
Arabidopsis thaliana	*fdh**	formate dehydrogenase mutant	*fdh*:D227Q/L229H (optimized)	atgcgtcaggcagcaaaagcaaccattcgtgcatgtagcagcagcagctcaagcggttattt<br>tgcacgtcgtcagtttaatgcaagcagcggtgatagcaaaaagattgttggtgttttctacaag<br>gccaacgaatacgcaaccaaaaatccgaattttctgggttgtgttgaaaatgcactgggtattc<br>gtgattggctggaaagccagggtcatcagtatattgttaccgatgataaagaaggtccggatt<br>gcgaactggaaaaacatattccggatctgcatgttctgattagcaccccgtttcatccggcatat<br>gtgaccgcagaacgtattaagaaagccaaaaatctgaaactgctgctgaccgcaggtattg<br>gtagcgatcatattgatctgcaggcagcagccgcagcaggtctgaccgttgccgaagttacc<br>ggtagcaatgttgttagcgttgcggaagatgaactgatgcgtattctgattctgatgcgcaatttt<br>gttccgggttataatcaggttgttaaaggcgaatggaatgttgccggtattgcatatcgtgcatat<br>gatctggaaggtaaaaccattggcaccgttggtgcaggtcgtattggtaaactgctgttacagc<br>gtctgaaaccgtttggttgtaatctgctgtatcatcagcgtcatcagatggcaccggaattagaa<br>aaagaaaccggtgccaaatttgtcgaagatctgaatgaaatgctgccgaaatgtgatgtgatt<br>gttattaacatgccgctgaccgagaaaacccgtggcatgtttaacaaagaactgattggcaa<br>actgaaaaagggtgtgctgattgttaataatgcacgtggtgcaattatggaacgtcaggccgtt<br>gttgatgcagttgaaagcggtcatattggttga

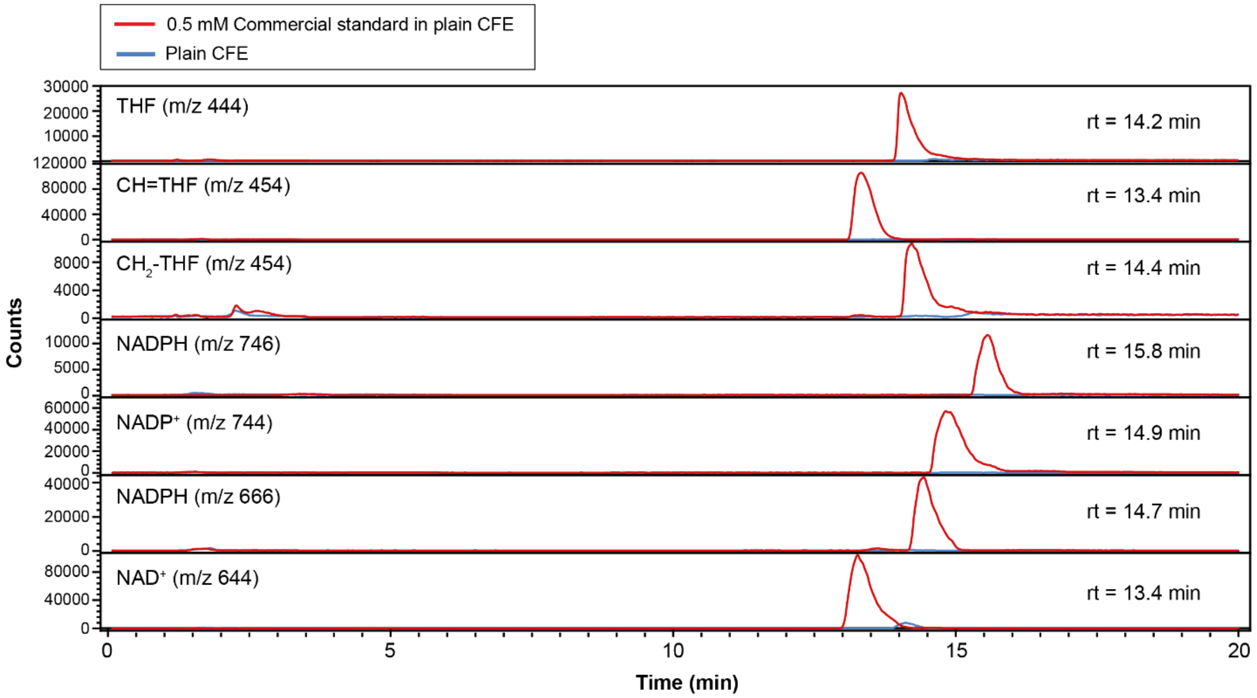

Figure S1. LC/MS traces of commercial tetrahydrofolate (THF), 5,10-methenyltetrahydrofolate (CH=THF), 5,10 methyleneltetrahydrofolate (CH_2-THF) in plain cell-free expression. Chemical were identified via single ion monitoring at the m/z specified. rt = retention time

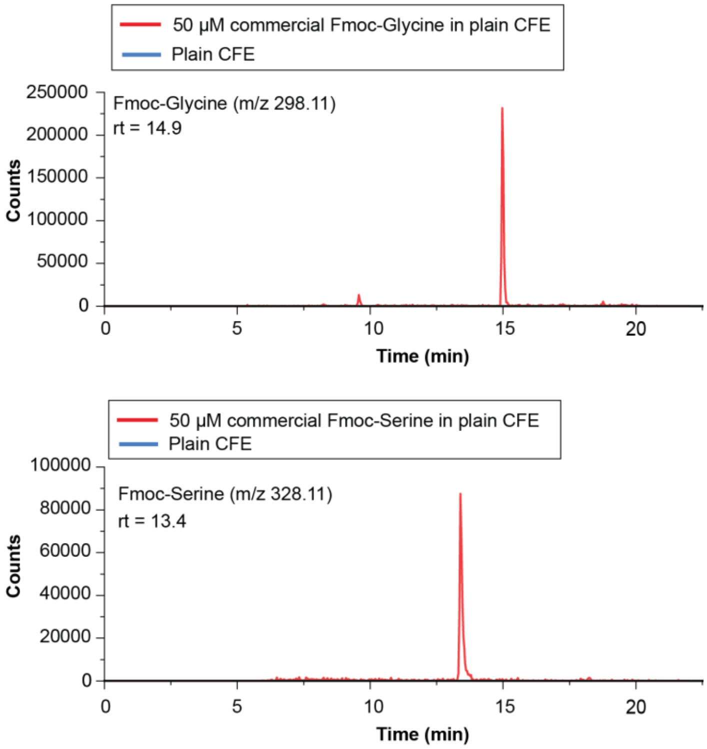

Figure S2. LC/MS traces of commercial Fmoc-Serine and Fmoc-glycine in plain cell-free expression (CFE). Chemical were identified via extracted ion chromatogram at the m/z specified. rt= retention time

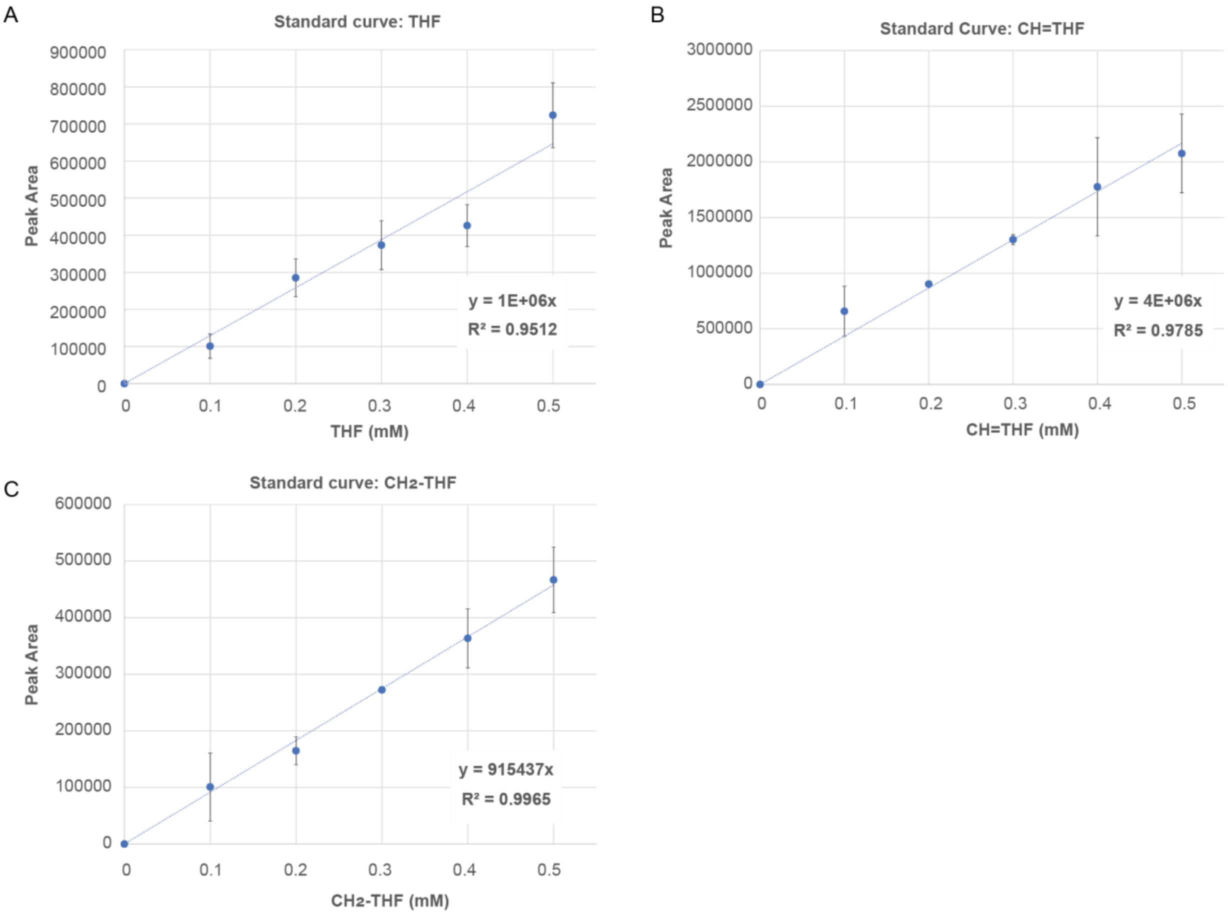

Figure S3. Standard curves using commercial tetrahydrofolate (THF), 5,10-methenyltetrahydrofolate (CH=THF) and 5,10 methyleneltetrahydrofolate (CH_2-THF).

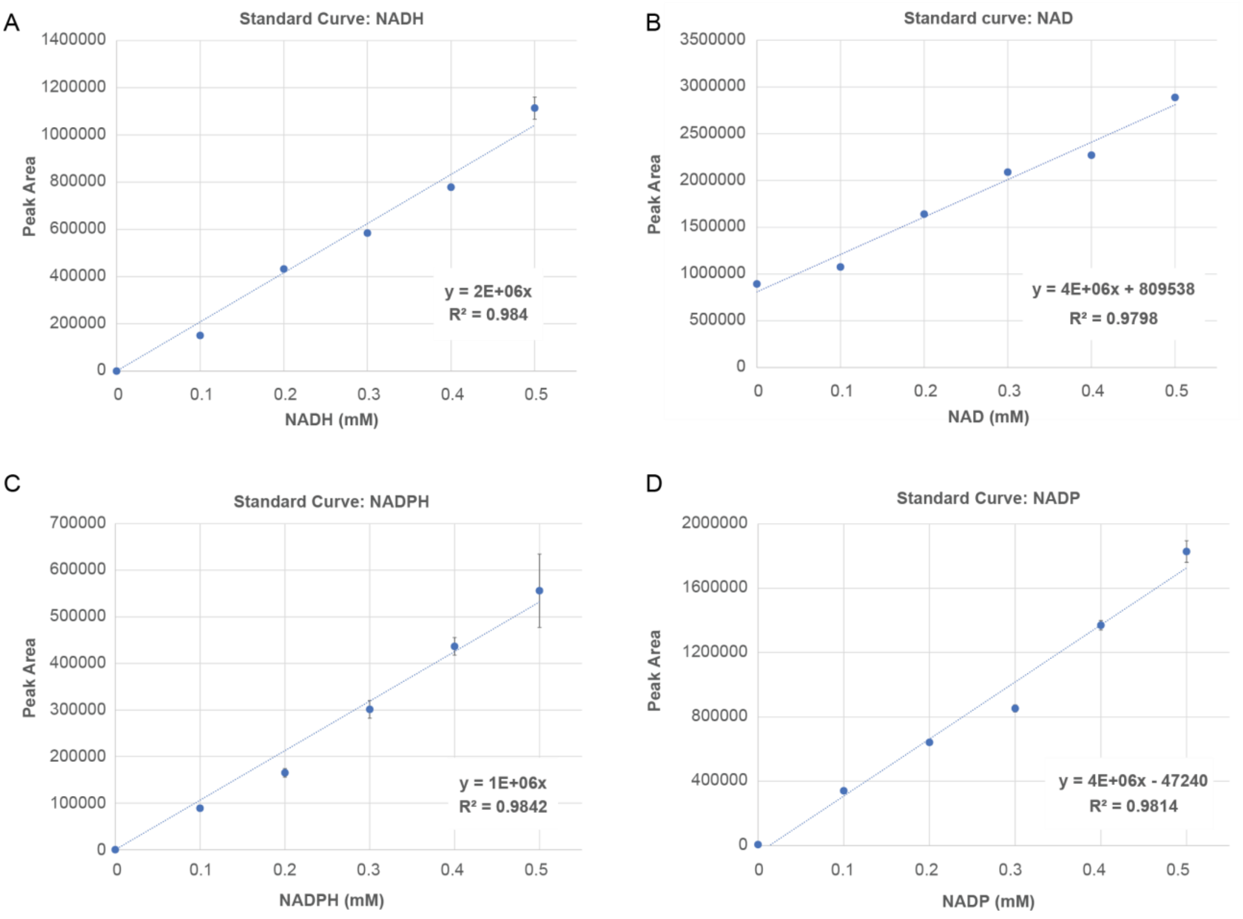

Figure S4. Standard curves using commercial NADH, NAD^+, NADPH, and $NADP^+$.

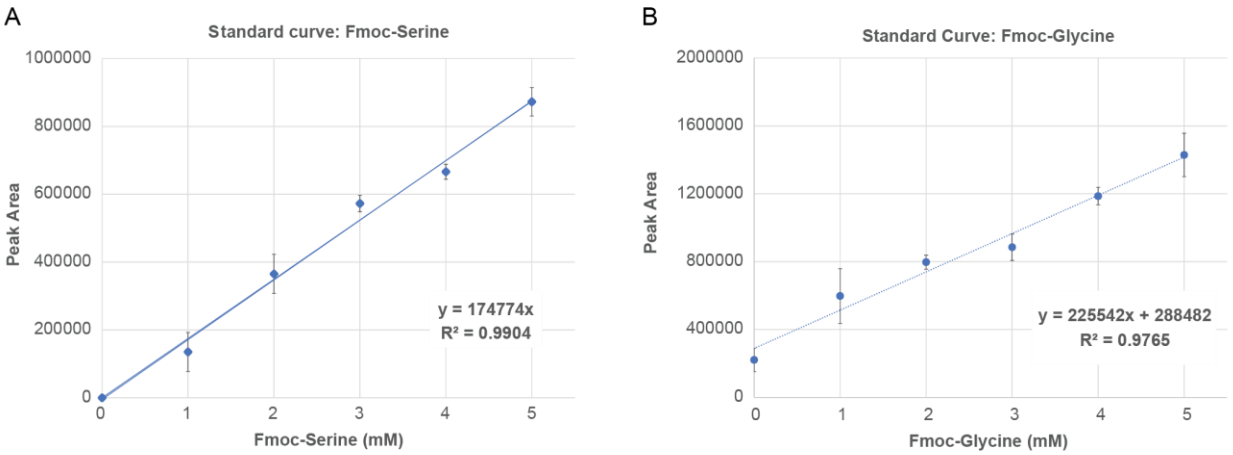

Figure S5. Standard curves of Fmoc-Serine and Fmoc-Glycine.

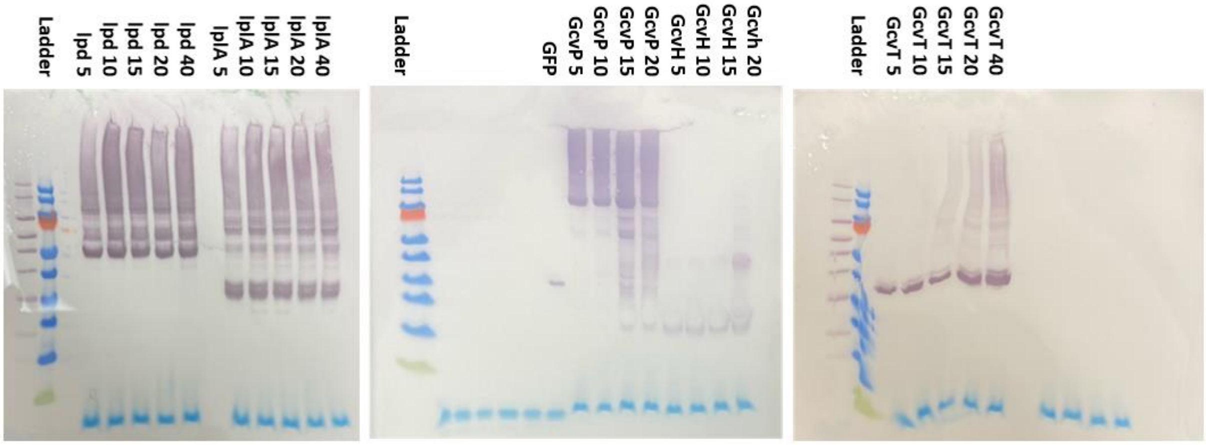

Figure S6. Complete Western blots Figure 4D

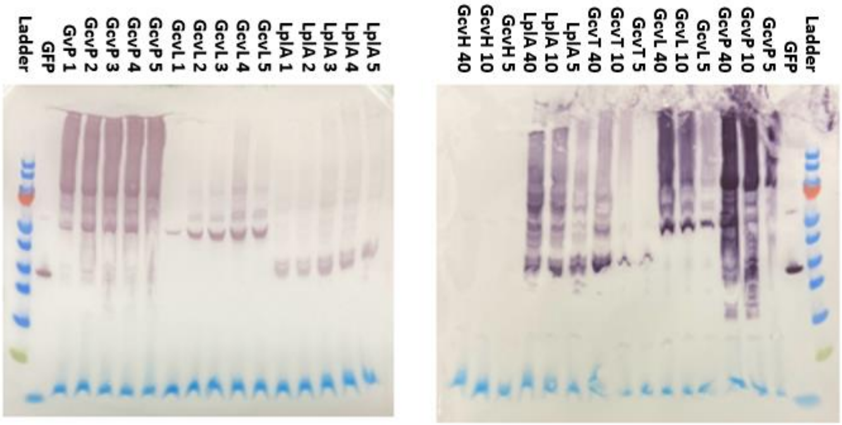

Figure S7. Complete Western blots Figure 4E

References

1. Zhou et al. Crystal structure of phosphite dehydrogenase provide insights into nicotinamide cofactor regeneration. Biochemistry, 2012, 51, 4263-4270.
